# Can Neonatal Systemic Inflammation and Hypoxia Yield a Cerebral Palsy-Like Phenotype in Periadolescent Mice?

**DOI:** 10.1007/s12035-019-1548-8

**Published:** 2019-04-02

**Authors:** Adamantia F. Fragopoulou, Yu Qian, Rochellys Diaz Heijtz, Hans Forssberg

**Affiliations:** 1 Department of Neuroscience, Biomedicum, Karolinska Institutet, 171 77 Stockholm, Sweden; 2 Department of Women’s and Children’s Health, Karolinska Institutet, 171 76 Stockholm, Sweden; 3 INSERM U1239, University of Rouen Normandy, 76130 Mont-Saint-Aignan, France

**Keywords:** Cerebral palsy, Systemic inflammation, Hypoxia, Behaviour, Motor skill learning, Microglia

## Abstract

Cerebral palsy (CP) is one of the most common childhood-onset motor disabilities, attributed to injuries of the immature brain in the foetal or early postnatal period. The underlying mechanisms are poorly understood, rendering prevention and treatment strategies challenging. The aim of the present study was to establish a mouse model of CP for preclinical assessment of new interventions. For this purpose, we explored the impact of a double neonatal insult (i.e. systemic inflammation combined with hypoxia) on behavioural and cellular outcomes relevant to CP during the prepubertal to adolescent period of mice. Pups were subjected to intraperitoneal lipopolysaccharide (LPS) injections from postnatal day (P) 3 to P6 followed by hypoxia at P7. Gene expression analysis at P6 revealed a strong inflammatory response in a brain region-dependent manner. A comprehensive battery of behavioural assessments performed between P24 and P47 showed impaired limb placement and coordination when walking on a horizontal ladder in both males and females. Exposed males also displayed impaired performance on a forelimb skilled reaching task, altered gait pattern and increased exploratory activity. Exposed females showed a reduction in grip strength and traits of anxiety-like behaviour. These behavioural alterations were not associated with gross morphological changes, white matter lesions or chronic inflammation in the brain. Our results indicate that the neonatal double-hit with LPS and hypoxia can induce subtle long-lasting deficits in motor learning and fine motor skills, which partly reflect the symptoms of children with CP who have mild gross and fine motor impairments.

**Electronic supplementary material:**

The online version of this article (10.1007/s12035-019-1548-8) contains supplementary material, which is available to authorized users.

## Introduction

Cerebral palsy (CP) is a common childhood motor disorder resulting in life-long functional limitations. The estimated prevalence in high-income countries is 1.8–2.3 cases per 1000 children [1–3], considerably higher in low- and middle-income countries [4]. The CP diagnosis includes several sub-types, i.e. spastic, ataxic and dyskinetic, and the severity of the motor symptoms ranges from light dyscoordination to severe gross and fine motor impairments hindering all independent daily activities. In addition, CP is often accompanied by sensory and cognitive dysfunctions and other medical conditions such as epilepsy.

Over time, a plethora of experience-derived treatments for CP have been used, and it is only during recent decades that there has been an evidence-based approach to identify and use interventions that effectively improve motor function and activity [5]. A common denominator for effective therapies is that they are all based on active motor learning and motor training [6–9]. Recent rodent studies have started to explore the molecular machinery of the learning-induced plasticity underlying motor learning, demonstrating the crucial involvement of dopamine signalling in the cortical-striatal circuitry [10–13]. So far, these studies have been performed in normal (uninjured) animals. In order to investigate the potential to use learning-induced plasticity to develop new interventions for CP, there is a need to develop a translational animal model that displays the motor phenotype of CP, i.e. the gross and fine motor impairments.

The aetiology of the brain injuries leading to CP varies and is multi-factorial, and it includes hypoxia-ischaemia (HI), infection and inflammation, and affects different brain areas depending on the age of the foetus/infant [14, 15]. Several animal models of CP have been suggested, including the well-known hypoxic-ischaemic Rice-Vannucci model [16–18]; various inflammation models (mimicking early infection), which mostly use the lipopolysaccharide (LPS; one of the major components of Gram-negative bacterial cell wall), or the interleukin 1b (IL1b) cytokine as inflammatory agents [19–23]; models that use hypoxia alone [24]; a combination of inflammation and HI [25–28]; and a combination of inflammation and hypoxia [29, 30]. In the majority of the studies, inflammation was induced prenatally, either alone [22] or in combination with HI postnatally [26, 31].

A limitation of the existing CP animal models is that they have primarily focused on the characterisation of the brain lesions and not on the functional consequences of these lesions (i.e. the CP phenotype) [32]. Moreover, the studies that have investigated behavioural outcomes have concentrated on the adulthood period [18, 33–35], while only a few studies have assessed the outcomes in young animals before sexual maturity [36, 21]. Furthermore, only a limited repertoire of behavioural functions has been investigated, e.g. there are no studies on fine motor skill performance.

Here, we examined the hypothesis that mice challenged neonatally with LPS and hypoxia would present behavioural deficits and brain tissue perturbations during the prepubertal to adolescent period that would resemble the CP phenotype in humans, using a comprehensive battery of behavioural tests, histological and molecular methods. Since CP is more common in males than in females [37] and different brain regions are affected depending mostly on the time and type of insult [38], we also intended to study whether LPS and hypoxia had a different impact between the sexes and whether different brain regions would react differently to this double insult.

## Materials and Methods

### Animals and Experimental Design

Pregnant C57BL/6J female mice (gestational day 13) were obtained from Janvier Laboratories (Le Genest-Saint-Isle, France) and housed individually in standard plastic cages (Makrolon Type III, Tecniplast, Buguggiate, Italy) under controlled temperature, humidity and light (12:12 h light-dark cycle) conditions. Food and water were supplied ad libitum. After birth (the day of birth was defined as postnatal day one, P1), pups were randomly assigned to either experiment A or B (see below). The treatment that followed (“LPS” or “LPS + Hypoxia”) did not affect the body weight of the dams or of their offspring (data not shown). In addition, differences in maternal behaviour were not observed.

#### Experiment A

The effect of single daily intraperitoneal (i.p.) injections of LPS (Sigma; 0.3–0.6 mg/kg; dose increasing day by day in order to prevent tolerance [39]) or of vehicle (sterilised water) during the first postnatal days (P3–P6) on the brain was studied. In both conditions—exposed to LPS (LPS) or control (Vehicle)—males and females were equally considered. Pups derived from 14 litters (7 Vehicle and 7 LPS) were used for the mortality rate calculation at P6. Some mice were decapitated 4 h after the last injection at P6, to examine whether the LPS initiated an inflammatory process in the brain. Specifically, we studied the gene expression levels of pro- and anti-inflammatory cytokines (interleukin 1beta, *Il1b*; interleukin 6, *Il6*; interleukin 10, *Il10*; interleukin 18, *Il18*; tumour necrosis factor alpha, *Tnfa*), complement system factors (alpha chain of complement C1q subcomponent, *C1qA*; beta chain of complement C1q subcomponent, *C1qB*; complement component 3, *C3*), pre-oligodendrocytes factors (oligodendrocyte transcription factor 1, *Olig1*; oligodendrocyte transcription factor 2, *Olig2*), myelin- and grey matter-associated genes (myelin basic protein, *Mbp*; myelin oligodendrocyte glycoprotein, *Mog*; microtubule-associated protein 2, *Map2*) and synaptic- and plasticity-related genes [synaptophysin, *Syp*; protein phosphatase 1 regulatory subunit 9B (spinophilin), *Ppp1r9b*; brain-derived neurotrophic factor, *Bdnf*] in both males and females (*n* = 6 mice/condition/sex derived from 3 to 4 litters per experimental group; 3 pups per litter were the maximum number of animals used per experimental group).

#### Experiment B

**Fig. 1:**
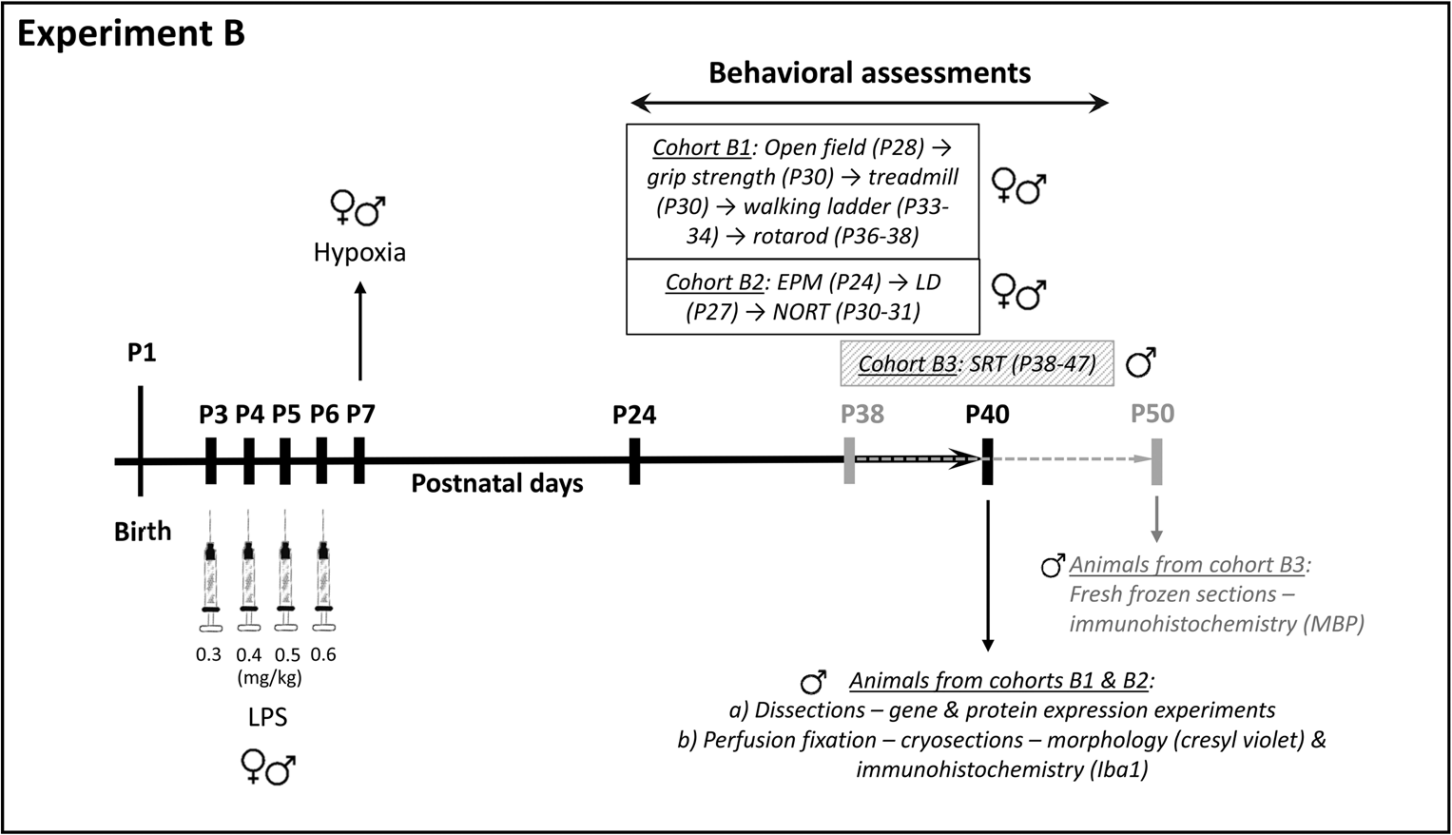
Schematic overview of the experimental design used in experiment B. Exposed mice were administered LPS from P3 to P6 and then subjected to hypoxia at P7 (24 h after the last LPS injection). Control mice were administered vehicle and then subjected to normoxia on the same dates. Mice were randomly assigned to three different cohorts (B1, B2 and B3) that were subjected to different behavioural tasks on the following days and order; cohort B1: open field (P28) → grip strength (P30) → treadmill (P30) → walking ladder (P33–34) → rotarod (P36–P38); cohort B2: EPM (P24) → LD (P27) → NORT (P30–31); cohort B3: SRT (P38–47). The number of animals used in each cohort was 13–19/condition/sex for cohort B1, except for the rotarod test, where 7–13 mice were used per condition and sex; 9–17 mice/condition/sex for cohort B2, and 5–6 male mice per condition for cohort B3. At P40, males from cohorts B1 and B2 were euthanised and their brains were processed for histological, immunohistochemical and molecular analysis (6 mice/condition/analysis), while at P50, mice from cohort B3 were euthanised and their brains were processed for immunohistochemical analysis (6 mice/condition). Abbreviations: *P* postnatal day, *LPS* lipopolysaccharide, *EPM* elevated plus maze, *LD* light/dark box, *NORT* novel object recognition task, *SRT* skilled reaching task, *MBP* myelin basic protein, *Iba1* ionised calcium binding adaptor molecule 1

Exposed mice were administered LPS (i.p.; from P3 to P6) as in experiment A, and then subjected to hypoxia (Biospherix chamber, 7 min ramp-up time until the chamber reached the 7.7% oxygen concentration, followed by 20-min inhalation of 7.7% oxygen-based nitrogen) at P7 (LPS + Hypoxia group). Control mice were administered vehicle (sterilised water) and then subjected to normoxia (22% oxygen, 27 min) on the same days (Vehicle + Normoxia group). Animals were kept undisturbed (except for the daily handling during P3 to P7, the weaning at P21 and the handling 3 days prior to the behavioural tasks) until used for functional assessment from P24 to P47 and subsequent brain tissue analysis (for a schematic overview of the study design, order of tests and number of animals, see Fig. 1). Three independent cohorts of animals were evaluated. In the first cohort (B1), locomotion, muscle strength, gait pattern and motor behaviour were tested (*n* = 13–19/condition/sex, except for the rotarod test where 7–13 mice were used per condition and sex). The reason why fewer animals were used in the rotarod is lost data due to technical problems where the last trials of some animals were not stored properly by the software. In the second cohort (B2), anxiety and cognition were evaluated (*n* = 9–17 mice/condition/sex), and in the third cohort (B3), motor skill learning was tested (males only; *n* = 5–6/condition). Mice of cohorts B1–B3 were derived from 21 litters in total; 10 litters were used for cohort B1 (5 Vehicle + Normoxia and 5 LPS + Hypoxia), 7 litters for cohort B2 (3 Vehicle + Normoxia and 4 LPS + Hypoxia) and 4 litters for cohort B3 (2 Vehicle + Normoxia and 2 LPS + Hypoxia). Male mice from cohorts B1 and B2 were sacrificed at the end of the behavioural tests at P40, either by cervical dislocation or deep anaesthesia and perfusion, and their brains were isolated for molecular (*n* = 6 per condition) or histological/immunohistochemical analysis (*n* = 6 per condition), respectively. Mice from cohort B3 were sacrificed at the end of the behavioural task at P50, by cervical dislocation, and their brains were isolated for immunohistochemical analysis (*n* = 6 per condition). The maximum number of animals per litter per experimental group for experiment B was 3 to 6 depending on the analysis (molecular/histological or behavioural).

### Behavioural Assessment

The three different cohorts of mice (B1, B2, B3) in experiment B were exposed to a battery of well-established behavioural tests from P24 to P47 (Fig. 1). All animals were naive to the tasks. Testing took place between 9.00 and 16.00 under low illumination to reduce stress. On the day of testing, animals were brought in their home cages to the testing room and allowed to rest for at least 1 h before testing. The order of tests was kept the same for all animals. All test chambers were cleaned with 70% ethanol and water after each animal.

#### Open Field

Animals were placed individually in the centre of an open-field box (48 cm × 48 cm; Acti-Mot detection system; TSE, Bad Homburg, Germany), and their spontaneous horizontal and vertical motor activity was recorded for 60 min, as previously described [40]. The computer programme automatically recorded the following parameters: total distance travelled, time spent in the centre and rearing activity.

#### Grip Strength

A validated grip strength test meter (BIOSEB, In Vivo Research Instruments, France) was used to measure the grip strength of the front limbs of the animals, as previously described [41]. Briefly, during the test, mice were handled by their tails and placed over the grid until the front paws grasped the grid. The tail was then pulled horizontally until the mouse released hold entirely. Five separate readings of the strength were recorded in grams (gr). Average and maximum values were evaluated.

#### Treadmill

The gait pattern was examined using ventral plane videography, as previously described [42]. The apparatus consisted of a motor-driven transparent treadmill belt (Exer Gait XL, Columbus Instruments, USA) and a high-speed digital video camera (100 fps) to record the ventral view of the treadmill belt, as reflected by an angled mirror. Mice were firstly habituated to the treadmill for 3 min, followed by 1 min during which the speed was progressively increased from 0 to 10 m/min. Once the testing speed was achieved, gait was recorded during three 20-s trials separated by 1-min intervals. The contacts made with the treadmill by each individual paw were determined using the TreadScan software (Treadscan 4.0, CleverSys, Inc., Reston, VA, USA). The following gait parameters were measured: percentage of swing (the percentage of the stride time during which the paw is in the air) and the inter-foot distance (foot spacing). The foot spacing was evaluated by four sub-parameters—front track width (FrTrWd): distance between the front two feet; rear track width (RrTrWd): distance between the rear two feet; left foot base (LeftFtBs): distance between the left feet pair; right foot base (RightFtBs): distance between the right feet pair.

#### Walking Ladder with Rungs

This test was performed according to previous studies [43–45]. The ladder rung apparatus was composed of two Plexiglas transparent walls (100 cm × 20 cm). Each wall contained holes, which could be filled with 3-mm-diameter metal bars, spaced 1 cm apart and from the bottom edge of the wall. The walls were spaced 3 cm apart to allow for passage of a mouse but preventing it from turning around. A 90 cm × 20 cm mirror was placed under the apparatus for angle view. Animals were tested in two ladder conditions for two consecutive days. Briefly, on the first day, mice were trained on a regular rung pattern for three trials in order to be able to walk the length of the alleyway without hesitation. The next day, rungs were removed in order to create an irregular pattern (same for all mice) within the alleyway. The distance between the metal rungs varied from 1 to 3 cm. Mice were allowed to cross the alleyway once. With the aid of the mirror positioned below the alleyway and a video camera, the lateral and ventral view of the mouse as it was crossing the rungs was recorded. Videos were manually analysed frame by frame, and the following parameters were scored: slight foot slips (error grade 1), deep foot slips (error grade 2) and total misses (error grade 3). Total score of errors = [(number of slight foot slips × 1) + (number of deep foot slips × 2) + (number of total misses × 3)].

#### Rotarod

The task was performed as previously described with some modifications [46] using a five-station rotarod treadmill (Ugo Basile, Italy). The day before testing, mice were acclimated to the apparatus by being placed on the cylinder at a rotating speed of 4 rpm for two 90-s periods, 2 h apart. The testing consisted of five trials per day, separated by 5 min each, over the course of 2 days (10 trials in total). Each trial ended when a mouse fell off, made one complete backwards revolution while hanging on, or reached 300 s. The first five trials took place under a standard range of acceleration (4 to 40 rpm), while in the last five trials, mice ran in more challenging conditions (acceleration speed 8 to 80 rpm). Time of fall was recorded, which is indicative of the animals’ gross motor coordination and balance.

#### Elevated Plus Maze

Mice were individually placed in the centre of an elevated plus maze apparatus (Kinder Scientific, Poway, CA, USA), facing an open arm, and allowed to explore for 5 min, as previously described [47]. The time spent in, and entries into, the open and closed arms, and total ambulation, were recorded via infrared photobeams and analysed with Motor Monitor software (Kinder Scientific, Poway, CA, USA).

#### Light/Dark Box

Mice were individually placed into the dark compartment and allowed to freely explore the apparatus (48 cm × 48 cm; with two zones of equal areas) for 5 min, as previously described [47]. Time spent in the dark and light compartments was measured by using photocells (TSE, Bad Homburg, Germany).

#### Novel Object Recognition Task

The task was performed as previously described with slight modifications [48]. The protocol we followed consisted of a habituation session, a familiarisation session and a probe trial. During the habituation session (day 1), each mouse was allowed to freely explore a 40 cm × 40 cm open arena for 15 min. The day after, the familiarisation phase took place first, where each mouse was allowed to explore two identical objects for 15 min. After an inter-trial interval of 30 min, each mouse was placed back for 5 min into the same arena, where one of the two familiar objects (randomly chosen each time) had been replaced by a new one of different shape, colour and texture (probe trial). The time spent exploring each object during the probe trial was scored and the preference index (time spent exploring the novel object/total exploration time) was calculated. An increased index was interpreted as enhanced performance.

#### Skilled Reaching Task

The skilled reaching task paradigm was performed as previously described [40]. We performed the task in males only since the oestrus cycle can affect plasticity and motor learning of female mice after P35. Prior to and during the training, mice were subjected to mild food deprivation. Mice underwent a 2-day pre-training period, the purposes of which were to introduce each animal to the training Plexiglas box (8 cm W × 21.5 cm L × 20 cm H) and to identify the preferred forelimb for reaching/grasping a 20-mg sweetened food pellet (5TUL, Cat. No 1811142; Test Diets, Indiana, USA) through a narrow opening in the centre of the box. During the subsequent 8 testing days, mice underwent a 20-min training session per day consisting of 30 discrete trials, to reach a food pellet and to grasp and retrieve the pellet with a single forelimb. Success was defined when an animal grasped a food pellet, transported it in the paw into the training box and placed it into its mouth. The following measurement was analysed: success per number of attempts % = (number of pellets obtained / number of attempts) × 100.

### Quantitative Real-Time Polymerase Chain Reaction

Mice were euthanised by decapitation at P6 (experiment A) or by cervical dislocation at P40 (experiment B) and brain tissues (prefrontal cortex, PFC; striatum, STR; hippocampus, HIP; cerebellum, CER) were rapidly isolated on ice, frozen in dry ice and stored at − 80 °C until further processing. Expression of genes of interest was quantitatively determined by using a SYBR Green Real-Time PCR Detection System (Bio-Rad, Sundbyberg, Sweden) as previously described [49]. The housekeeping gene TATA-box binding protein (*Tbp*) was used for normalisation, as its expression was stable throughout postnatal development in all tissues examined. The Primer-BLAST web-based software was used to design gene-specific primers (http://www.ncbi.nlm.nih.gov/tools/primer-blast/). All primer sequences are listed in Suppl. Table 1.

### Western Blot Analysis

Western immunoblotting was performed as previously described [11]. The membranes were immunoblotted using the rabbit monoclonal anti-spinophilin antibody (cat no. 14136, 1:10,000, Cell Signalling Technology, Inc.). The mouse monoclonal antibody against GAPDH (cat no. ab8245, 1.10,000, Abcam) served as loading control. Antibody binding was revealed by incubation with horseradish peroxidase-conjugated secondary antibodies (anti-rabbit, Cat. No 170-5046, 1:5000, Bio-Rad and anti-mouse, Cat. No 170–5047, 1:5000) and Clarity™ western ECL substrate (Bio-Rad). Protein band detection and quantification were carried out using the ChemiDoc™ XRS+ System with Image Lab™ Software (Bio-Rad, Sundbyberg, Sweden).

### Histology–Immunohistochemistry and Image Data Analysis

Mice from cohorts B1 and B2 were anaesthetised at P40 with an i.p. injection of sodium pentobarbital (100 mg kg^−1^ of body weight; Apoteket, Stockholm, Sweden) and perfused via the ascending aorta with a mixture of paraformaldehyde and picric acid [4% paraformaldehyde and 0.2% picric acid, in 0.01 M phosphate-buffered saline (PBS), pH 7.35]. The brains were rapidly dissected, immersed in the same fixative for at least 4 h and then cryoprotected in 10% sucrose, 0.02% bacitracin (Sigma) and 0.01% sodium azide (Sigma), in PBS for at least 36 h. Brains were subsequently frozen in isopentane (− 50 °C), dry ice and then stored at − 80 °C until further processing. Brain sections were cut at 20 μm using a cryostat, collected on SuperFrost Plus slides (VWR, Stockholm, Sweden) and stored at − 20 °C until additional processing.

For the histological analysis, coronal sections corresponding to 0.86–1.18 mm from bregma [50] were proceeded for cresyl violet staining. After warming the frozen sections at room temperature, fixation with 4% paraformaldehyde (PFA, Cat. No. P6148, Sigma-Aldrich) and rinsing with water followed. Next, cresyl violet staining was performed (0.5% *w*/*v* Cresyl violet acetate, Sigma), then washes in water, hydration of the tissue sections with graded alcohol series and xylene, and finally cover-slipping with DPX mounting medium (VWR, England). The sections were analysed microscopically under a bright-field microscope (Olympus BH2) connected to an SC30 camera (Olympus Sverige, Stockholm, Sweden). Area measurements were performed using the Cell Sens Imaging Software ver. 1.12 (Olympus, Tokyo, Japan).

For the immunofluorescence analysis, sections were immunostained, with a goat polyclonal antibody against Iba1 (ionised calcium binding adaptor molecule 1) to label microglial cells (1:800, 21-h incubation; Cat. No. ab5076, Abcam), as previously described [47]. Signal detection was achieved using Donkey Anti-Goat Cy5 antiserum (1:250, 2 h; Jackson ImmunoResearch Europe, Suffolk, UK). Photomicrographs were taken with an Olympus BX53 microscope connected to an XM10 camera (Olympus Sverige, Stockholm, Sweden). Digital camera illustrations were prepared using the Cell Sens Imaging Software ver. 1.12 (Olympus, Tokyo, Japan). Images were only adjusted for brightness and contrast. Microglia density was quantified using the Image J software (v.1.46r, NIH, USA) after segmenting the images by the background and threshold for the immunostaining applied.

Mice of cohort B3 were sacrificed by cervical dislocation at P50 and their brains were isolated and snap frozen in isopentane and dry ice and then stored at − 80 °C until further processing. Brain sections were cut at 10 μm using a cryostat, collected on SuperFrost Plus slides and stored at − 20 °C until additional processing. Myelin basic protein (MBP) immunohistochemical analysis was performed as previously described [51] using the mouse monoclonal antibody against MBP (1:1000, 30-min incubation; Cat. No SMI-99, Covance, NJ, USA), the Vector® Mouse on Mouse (M.O.M.™) Basic Immunodetection Kit (Cat. No BMK-2202, Vector Laboratories, Sweden) and the VECTASTAIN® Elite ABC-Peroxidase Kit according to the manufacturers’ specifications. The enzymatic coloration of immunoreactivity was performed by 3,3′-diaminobenzidine, DAB (DAB staining kit, Cat. No K3468, Agilent, CA, USA). Photomicrographs were taken with a Zeiss Axio Imager M2 microscope (Zeiss, Oberkochen, Germany) connected to a Lumina HR Camera (MBF, Williston, VT, USA). Images were captured by using the Stereology Investigator software (MBF) and were only adjusted for brightness and contrast. MBP area and optical density (OD) measurements were taken in the centre of corpus callosum and the cingulate cortex and were performed using the Image J software.

### Statistical Analysis

Statistical analysis was performed using SPSS v.24 software (SPSS Inc., Chicago, IL, USA). Results were expressed as means ± standard error of the mean (SEM). The Shapiro–Wilk test was used to assess the normality of the data and the Levene’s test to confirm homogeneity of variances. For the mortality rate, Chi-square analysis was performed. For the molecular and behavioural experiments, two-way analysis of variance (ANOVA) was used to reveal any condition (LPS or LPS/hypoxia) effect or sex effects, or condition × sex interaction, with “condition” and “sex” as between-subject factors. For the behavioural assessments, to test the “condition” effect between the exposed and the control groups within the same sex, either repeated-measure ANOVA, independent sample *t* test or Mann–Whitney *U* test were used when appropriate. Post hoc comparisons were made using a Bonferroni test when significant ANOVA effects were found. Data from gene expression experiments were analysed using the Mann–Whitney *U* test followed by false discovery rate (FDR) correction for multiple comparisons. Data from Western blot and immunohistochemistry experiments were analysed by the Mann–Whitney *U* test. The threshold for statistical significance was set as *p ⩽* 0.05.

## Results

### Experiment A

#### LPS Induces Acute Brain Inflammation and Alterations in the Expression of Synaptic and Plasticity-Related Genes in both Male and Female Mice

**Fig. 2:**
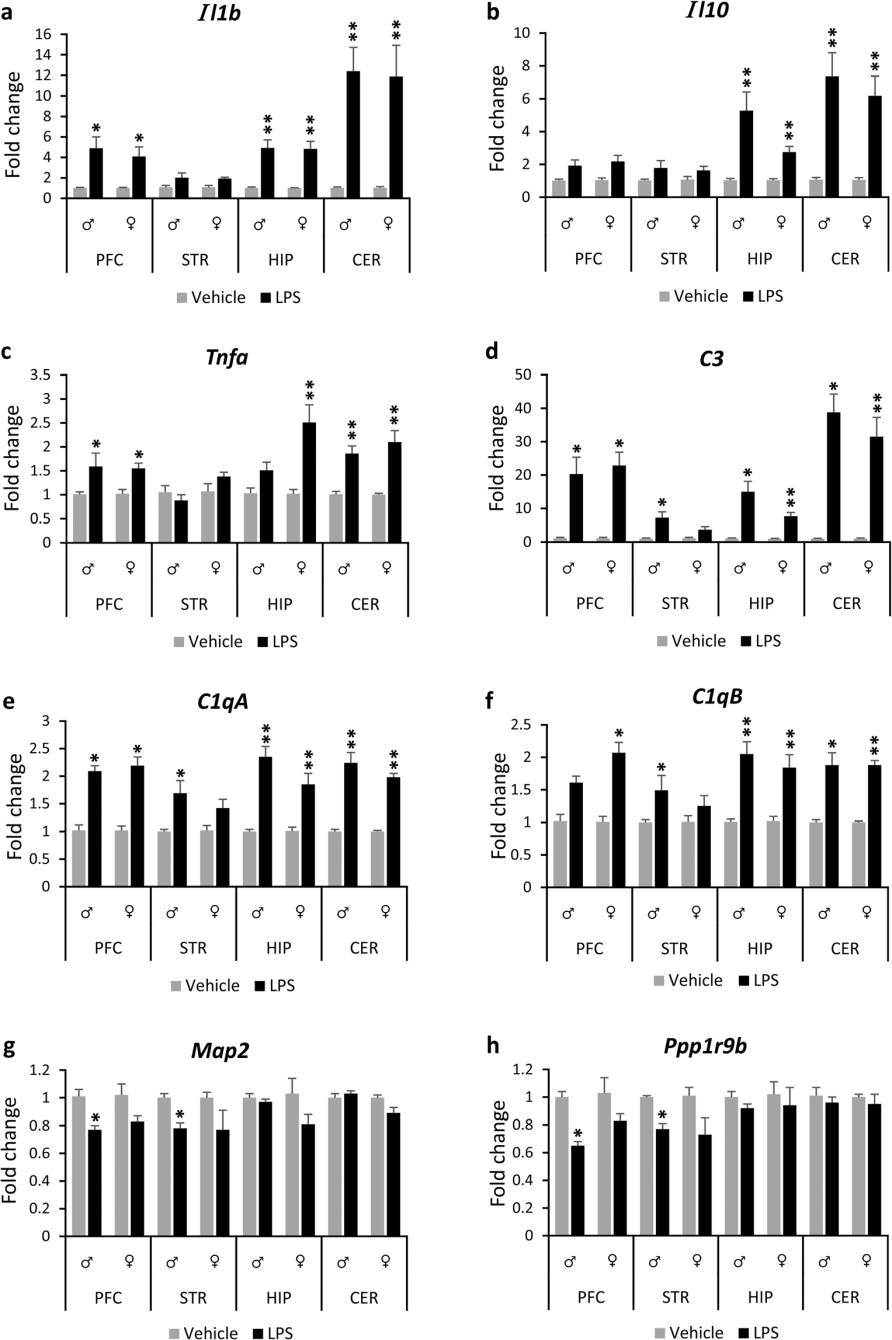
Brain region-specific changes in gene expression at P6 in response to neonatal LPS challenge. Bar graphs show gene expression levels of cytokines (*Il1b*, *Il10*, *Tnfa*; **a**–**c**), complement system factors (*C1qA*, *C1qB*, *C3*; **d**–**f**) and neuronal markers (*Map2*, *Ppp1r9b*; **g**, **h**) in the prefrontal cortex, striatum, hippocampus and cerebellum of exposed male and female mice and their respective controls. All data (**a**–**h**) are presented as means (± SEM). (*n* = 6/condition/sex). *p < 0.05, **p < 0.01 when compared with their respective control groups. Abbreviations: *Il-1b* interleukin 1beta, *Il10* interleukin 10, *Tnfa* tumour necrosis factor alpha, *C1qA* alpha chain of complement C1q subcomponent, *C1qB* beta chain of complement C1q subcomponent, *C3* complement component 3, *Map2* microtubule-associated protein 2, *Ppp1r9b* protein phosphatase 1 regulatory subunit 9B, *PFC* prefrontal cortex, *STR* striatum, *HIP* hippocampus, *CER* cerebellum, *LPS* lipopolysaccharide

In the first experiment, we investigated whether the selected LPS protocol induced inflammation in the brain, and therefore, it could be used as the first insult of a double hit model in experiment B. The mRNA levels of *Il1b*, *Il10* and *Tnfa* were significantly (*p* < 0.05) upregulated (e.g. ranging from 1.5- to 12-fold) at P6 in the cerebellum, hippocampus and prefrontal cortex regions of both male and female mice exposed to LPS from P3 to P6 compared to their respective controls with minor exceptions (Fig. 2, see Suppl. Tables 2, 3, 4 and 5 for the Mann-Whitney *U* and *p* values). The expression levels of *Il10* did not change in the prefrontal cortex of the exposed males and females, and the *Tnfa* levels did not change in the hippocampus of the exposed males (*p* > 0.05)*.* No changes were observed in the mRNA levels of *Il1b*, *Il10* and *Tnfa* in the striatum of the exposed males or females compared to their controls. In addition, there was a 2.5-fold increase in the mRNA levels of *Il6* in the cerebellum (*p* = 0.005, Suppl. Table 5) and a small but significant reduction in the mRNA levels of *Il18* in the prefrontal cortex (17%) and hippocampus (26%) of the exposed females compared to their controls, but not in the exposed males (Suppl. Tables 2 and 4).

The mRNA levels of the complement system factors *C3*, *C1qA* and *C1qB* were substantially (*p* < 0.05) increased (e.g. ranging from 1.5-fold to 38-fold) in all the brain regions studied of the exposed males and females, except the case of the mRNA levels of *C1qB* in the prefrontal cortex of males and the *C3*, *C1qA* and *C1qB* in the striatum of females, which did not change between the respective control and exposed groups (Fig. 2, Suppl. Tables 2, 3, 4 and 5). In contrast, the mRNA levels of *Map2* and *Ppp1r9b* were significantly decreased in the prefrontal cortex (24% and 35% decrease, respectively) and striatum (22% and 23% decrease, respectively) of the exposed male mice compared to their controls. A similar pattern of expression was found in the prefrontal cortex and striatum of the exposed females, but did not reach statistical significance (*p* > 0.05) (Fig. 2, Suppl. Tables 2 and 3). In addition, there was a small but significant reduction in the expression of *Olig1* in the prefrontal cortex (11%) and hippocampus (12%) of the exposed females, but not in the exposed males (Suppl. Tables 2 and 4). Moreover, *Bdnf* mRNA levels were also decreased (21%) in the hippocampus of the exposed females compared to their respective controls (*p* < 0.05), while no change was observed in the exposed males (Suppl. Table 4).

The two-way ANOVA indicated a significant main interaction between condition and sex, only for *Tnfa* (*F*_(1, 19)_ = 4.966, *p* = 0.038) and *Il18* (*F*_(1, 20)_ = 8.802, *p* = 0.008) in the hippocampus, and not for the rest of the genes, where a main effect for condition was found. This indicates that any difference in the genes’ response was mainly dependent upon which substance was administrated (LPS or vehicle) and not on the sex of the animals.

Western blot *analysis* of cerebellar tissue samples at P6, which had the most robust inflammatory response at the gene expression level at this time point, showed a significant reduction in spinophilin protein levels in the exposed males compared to their controls (Suppl. Fig. 1a, b).

In conclusion, the first experiment showed that the LPS injections induced altered gene expression and an acute inflammation in a brain region-dependent manner.

#### Neonatal Systemic Inflammation Is Not Lethal

LPS alone had no effect on the survival rates of the animals under the specific conditions used in this study (dose, age of animals, method of administration), since none of the exposed animals died during the daily administration of LPS from P3 to P6. No control animal died after the injections with vehicle.

### Experiment B

#### The Combination of LPS and Hypoxia Leads to Increased Mortality

Systemic inflammation induced by LPS from P3 to P6, in combination with subsequent hypoxia at P7, had a significant effect on the survival rates of the animals (*χ*^2^ = 13.21, *df* = 1, *p* < 0.001). Sixteen out of 85 exposed to LPS and hypoxia animals died (18.8% mortality rate; 8 males out of 43 males and 8 females out of 42 females) during or immediately after the exposure to hypoxia. From the 76 control animals (38 males and 38 females), only one control male died after the treatment (i.e. vehicle injections followed by normoxia).

#### Neonatal Challenge with LPS and Hypoxia Induces Subtle Behavioural Alterations in Prepubertal and Adolescent Mice

##### Locomotion

**Fig. 3:**
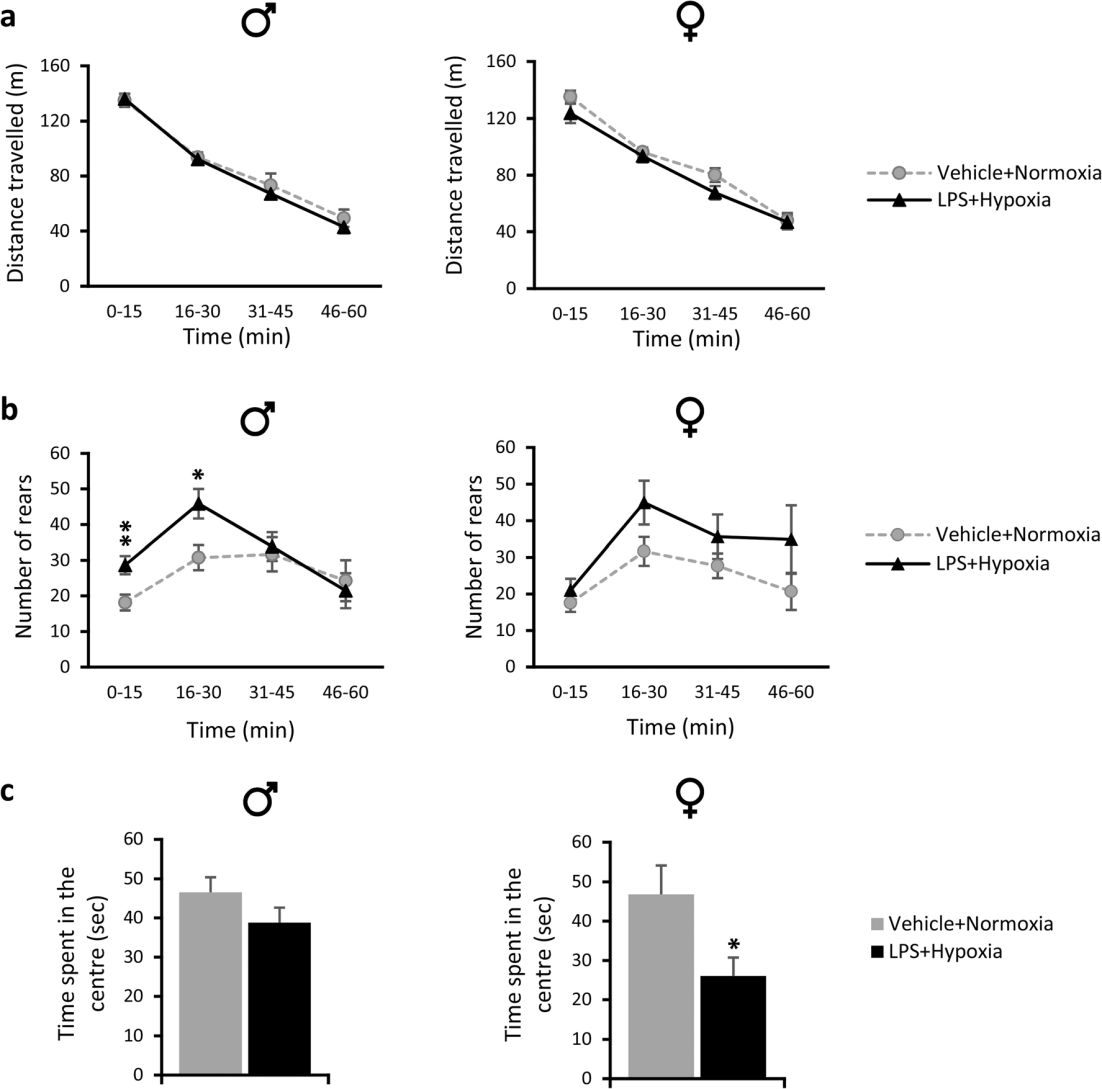
Performance of adolescent (P28) mice in the open-field test after the neonatal double hit of LPS and hypoxia. **a**, **b** Lines show the average distance travelled (m) (**a**) and the average number of rears (**b**) in 15-min time bins across a 60-min session in an open-field test by exposed male and female mice (black triangles) and their respective control groups (grey circles). **c** Bars show the time spent in the centre (sec) of the open-field box during the first 15 min of testing by exposed male and female mice (black bars) and their respective control groups (grey bars). All data are expressed as means ± SEM (*n* = 13–19/condition/sex). *p < 0.05, **p < 0.01 compared to their respective control groups. Abbreviation: *LPS* lipopolysaccharide

The exposed male and female mice did not show any changes in locomotion compared to their respective controls in the open-field test, as the distance travelled across time (time points of 15 min) was similar between the exposed and their respective control groups (*p* > 0.05; Fig. 3a).

##### Anxiety-Like Behaviour and Exploratory Activity

The exposed males spent more time in the open arms of the elevated plus maze (EPM) compared to their respective controls, less time in the closed arms and more time in the centre of the apparatus (*p* < 0.001, *p* < 0.001, and *p* = 0.025, respectively, Table 1). This shows that the exposed males were less anxious compared to their controls and/or that they displayed higher levels of exploratory behaviour. Increased exploratory activity and/or escape behaviour of the males were also demonstrated in the open-field test, where the exposed group had higher numbers of rears compared to their respective controls (repeated measures ANOVA for rearing activity across time; condition × time interaction *F*_(3,96)_ = 3.619, *p* = 0.016). Pairwise post hoc comparison revealed that there was a significant difference during the novelty phase (0–15 min: 57.9% increase in the number of rears in the exposed group compared to control, *p* = 0.005; 16–30 min: 49.2% increase in the exposed group compared to control, *p* = 0.011), but not during the habituation phase (31–45 min, *p* > 0.05 and 46–60 min, *p* > 0.05) of the open-field test (Fig. 3b). No signs of anxiety were shown for the exposed males compared to their respective controls in either the open-field or the light-dark (LD) test, as judged by the similar time spent in the centre of the open field and the similar time spent and distance travelled in the light and dark compartments of the LD box (*p* > 0.05, Table 1, Fig. 3c).

**Table 1:** Behavioural phenotype of prepubertal and adolescent male and female mice after a neonatal inflammation/hypoxia insult

			Males			Females		
Function	Behavioural test	Parameter studied	Veh + Norm	LPS + Hyp	*p* value	Veh + Norm	LPS + Hyp	*P* value
Anxiety-like behaviour and exploratory activity	*Elevated plus maze*	Time spent (s) - open arms	89.44 ± 4.70	119.72 ± 5.31	*< 0.001*	97.47 ± 9.27	85.44 ± 6.01	0.675
		-closed arms	174.65 ± 4.45	133.95 ± 5.69	*< 0.001*	166.23 ± 9.46	171.41 ± 6.40	0.863
		-centre	35.91 ± 1.97	46.31 ± 3.44	*0.025*	36.29 ± 1.68	43.14 ± 3.28	0.824
		Number of entries - open arms	22.57 ± 1.41	23.46 ± 2.09	1.000	24.10 ± 2.66	25.94 ± 1.65	0.170
		- closed arms	19.86 ± 1.41	20.08 ± 1.64	0.793	20.40 ± 1.44	20.53 ± 1.31	0.443
	*Light/dark box*	Time spent (s) - light	85.64 ± 9.02	89.79 ± 7.21	0.731	86.73 ± 11.78	83.65 ± 5.97	0.798
		-dark	214.46 ± 9.01	210.29 ± 7.21	0.729	213.34 ± 11.78	216.61 ± 5.94	0.785
		Distance travelled (m) - light	16.53 ± 1.38	16.25 ± 1.14	0.884	15.45 ± 2.34	18.91 ± 1.24	0.163
		-dark	35.01 ± 2.17	34.68 ± 2.91	0.927	35.20 ± 2.42	42.24 ± 2.16	*0.047*
Muscle strength	*Grip strength*	Average forelimb force (g)	60.59 ± 1.44	60.20 ± 1.87	0.867	69.98 ± 2.26	62.82 ± 1.55	*0.015*
		Maximum forelimb force (g)	76.55 ± 1.82	73.35 ± 2.73	0.338	83.11 ± 2.53	78.50 ± 3.28	0.278
		Animal weight (g)	15.19 ± 0.21	14.73 ± 0.25	0.161	13.77 ± 0.21	13.88 ± 0.18	0.682
Gait	*Treadmill*	% Swing - Front right	52.72 ± 0.73	53.78 ± 0.52	0.237	53.25 ± 0.55	54.21 ± 0.78	0.329
		- Front left	55.72 ± 1.06	57.93 ± 1.50	0.264	55.66 ± 1.31	56.02 ± 1.88	0.837
		- Rear right	40.76 ± 0.74	42.33 ± 0.87	0.197	38.91 ± 1.06	40.64 ± 1.10	0.222
		- Rear left	47.31 ± 0.95	49.79 ± 1.36	0.166	45.30 ± 1.11	45.86 ± 2.61	0.752
		Foot spacing (mm) - FrTkWd	11.39 ± 0.19	10.75 ± 0.16	*0.016*	11.10 ± 0.19	10.76 ± 0.23	0.268
		- RrTkWd	20.70 ± 0.37	19.55 ± 0.33	*0.026*	18.71 ± 0.33	19.09 ± 0.46	0.505
		- LeftFtBs	32.88 ± 0.23	33.00 ± 0.74	0.875	32.49 ± 0.92	32.23 ± 0.70	0.819
		- RightFtBs	32.76 ± 0.36	31.81 ± 0.57	0.197	32.41 ± 0.32	31.78 ± 0.74	0.441
Motor coordination and balance	*Walking ladder*	Score of errors	11.00 ± 1.40	15.25 ± 1.16	*0.029*	10.08 ± 1.11	15.08 ± 1.20	*0.006*
	*Rotarod*	Mean latency to fall on day 1 (s)	159.40 ± 15.09	151.81 ± 8.51	0.438	166.74 ± 15.81	148.12 ± 12.36	0.375
		Mean latency to fall on day 2 (s)	91.71 ± 9.50	98.23 ± 6.94	0.588	110.94 ± 8.39	119.51 ± 9.32	0.554
Object recognition	*NORT*	Preference index (%)	69.76 ± 4.25	65.21 ± 5.03	0.497	72.77 ± 4.49	66.51 ± 1.95	0.227

All data are presented as means ± SEM. Italics are statistically significant data

*NORT* novel object recognition task, *FrTrWd* front track width (distance between the front two feet), *RrTrWd* rear track width (distance between the rear two feet), *LeftFtBs* left foot base (distance between the left feet pair), *RightFtBs* right foot base (distance between the right feet pair), *Veh* vehicle, *Norm* normoxia, *LPS* lipopolysaccharide, *Hyp* hypoxia

Increased anxiety levels were observed for the exposed females in the open-field test since they spent 44.3% less time in the centre of the arena during the first 15 min of the task compared to their controls (*t*_(1, 24)_ = 2.379, *p* = 0.026; Fig. 3c). In addition, exposed females showed also signs of increased anxiety in the LD test, since they travelled more distance in the dark compared to their respective controls (*t*_(1, 25)_ = 2.088, *p* = 0.047; Table 1). However, since no changes were observed between the female exposed and control group in the time spent in the light and dark compartment of the LD test, or in the time spent and number of entries in the open and closed arms of the EPM test (*p* > 0.05, Table 1), then any anxiety observed in the open-field and LD can be considered as mild. The exposed females displayed a similar trend to the males for increased exploratory activity in the open-field task, but it did not reach significance (*p* > 0.05) (Fig. 3b). Moreover, no differences in exploratory activity were observed in the EPM, as judged by the time spent in the open arms (*p* > 0.05; Table 1).

Hence, males exposed to LPS and hypoxia displayed increased exploratory activity and exposed females showed traits of increased anxiety-like behaviour.

##### Muscle Strength

The exposed females showed a 10.2% reduction in the average forelimb strength compared to their controls (*t*_(1, 24)_ = 2.610, *p* = 0.015; Table 1), but no significant reduction in the maximum forelimb strength, while the forelimb grip strength (average and maximum) did not differ between the exposed and control male groups (*p* > 0.05; Table 1). Notably, the average body weight of the exposed and control groups within each sex was similar (*p* > 0.05; Table 1). Thus, only the exposed females showed a decrease in the average forelimb strength.

##### Gait Pattern

In the gait analysis, exposed male and female mice showed similar swing time compared to their respective controls (*p* > 0.05). The distance between the front paws and rear paws was slightly but significantly reduced in the exposed male group compared to their controls (FrTkWd: 5.6% reduction, *t*_(1, 32)_ = 2.537, *p* = 0.016; RrTkWd: 4.8% reduction, *t*_(1, 32)_ = 2.328, *p* = 0.026, respectively; Table 1), but not in the exposed female group (*p* > 0.05; Table 1). Thus, only exposed males showed minor alterations to foot placement during locomotion.

##### Motor Coordination and Balance

In the walking ladder test, both exposed male and female mice showed a significantly higher number of errors compared to their respective controls (males: 38.6% increase in the error score, *t*_(1, 22)_ = 2.338, *p* = 0.029; females: 49.6% increase in the error score, *t*_(1, 23)_ = 3.061, *p* = 0.006; Table 1). No significant differences were found in the rotarod test in any of the two acceleration conditions used (less and more challenging), as judged by the latency to fall over the 10 different trials (*p* > 0.05), or by the mean latency to fall per day (*p* > 0.05; Table 1). Hence, both exposed males and females had deficits in skilled walking, limb placement and limb coordination compared to their respective controls, but not in gross motor coordination and balance.

##### Object Recognition

In the novel object recognition task, there were no significant differences in the preference index between exposed and control animals in any sex (*p* > 0.05; Table 1). Thus, both exposed and control groups were able to recognise successfully the novel versus the familiar object, implying no effect on the recognition memory.

##### Motor Skill Learning and Performance

**Fig. 4:**
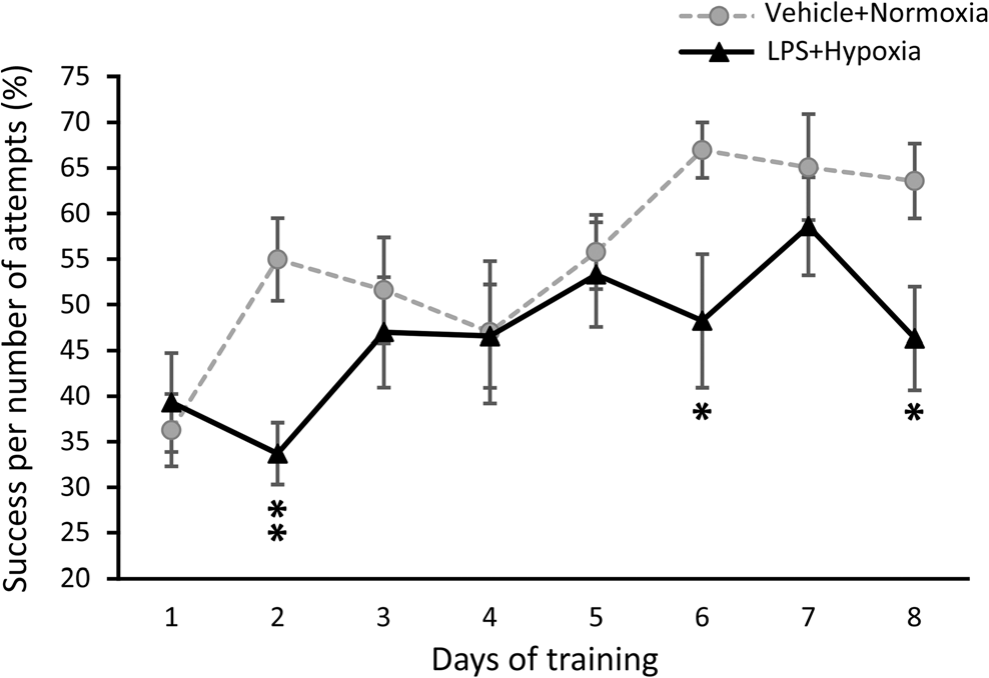
Daily performance of adolescent (P38–47) male mice in the skilled reaching task (SRT). The line graph shows the percentage of success per number of attempts in the 8-day training period of the SRT by exposed to LPS and hypoxia male mice (black triangles) and their respective control group (grey circles). Data are expressed as means ± SEM (*n* = 5–6/group). *p < 0.05, **p < 0.01 compared to the control group. Abbreviation: *LPS* lipopolysaccharide

The performance of the exposed and control male mice in the skilled reaching task is shown in Fig. 4. Repeated measures ANOVA for the percentage of successful reaches per number of attempts showed significant effects of condition (*F*_(1, 9)_ = 5.734, *p* = 0.04) and day of training (*F*_(7, 63)_ = 4.761, *p* < 0.001). The control mice improved their performance throughout the 8-day training period, as judged by the increased number of success per number of attempts per day (*F*_(7, 35)_ = 5.192, *p* < 0.001); thus, they were able to learn the task. In contrast, the exposed mice did not display any significant improvement over the 8-day training period (*F*_(7, 28)_ = 1.814, *p* > 0.05). Post hoc Bonferroni analysis confirmed that the exposed mice had significantly lower success scores than the controls (i.e. success per number of attempts (%)), especially on days 2, 6 and 8 (*p* = 0.006, *p* = 0.032 and *p* = 0.033, respectively). Thus, exposed males showed deficits in both motor skill learning and motor skill performance.

##### Sex Differences at the Behavioural Level

Two-way ANOVA showed a statistically significant interaction between “condition” and “sex” in the following parameters: (1) the time spent in the open arms of the EPM (*F*_(1, 50)_ = 11.226, *p* = 0.002) and the time spent in the closed arms of the EPM (*F*_(1, 50)_ = 12.310, *p* = 0.001) and (2) the distance between the rear paws in the treadmill (*F*_(1, 56)_ = 4.174, *p* = 0.046). Overall, this analysis indicates that sex differences at the behavioural level were restricted to only a few parameters of testing. Notably, since exposed to LPS and hypoxia males showed gait alterations and fine motor skill deficits, which are important CP symptoms, only the male brains were further analysed at P40 and P50.

#### Neonatal Challenge with LPS and Hypoxia Does Not Lead to Major Changes in Gene Expression in the Brains of Adolescent Male Mice

Gene expression analysis of pro- and anti-inflammatory cytokines (*Il1b*, *Il10*, *Tnfa*), complement system factors (*C1qA*, *C1qB* and *C3*), myelin (*Mbp* and *Mog)*, grey matter (*Map2*), synaptic (*Syp* and *Ppp1r9b*) and plasticity (*Bdnf*) related genes did not reveal any major changes in the brain regions (prefrontal cortex, striatum, hippocampus, cerebellum) of adolescent male mice exposed neonatally to LPS and hypoxia compared to their controls (*p* > 0.05; see Suppl. Table 6 for Mann-Whitney *U* and *p* values). Only two genes (*C1qB* and *Bdnf*) were differentially expressed between the exposed and control groups. The mRNA levels of *C1qB* were significantly decreased in the prefrontal cortex (31%) and hippocampus (28%), changes that are in the opposite direction to earlier results for younger animals after LPS exposure, while *Bdnf* expression levels were increased (1.35-fold) in the cerebellum (*p* < 0.05; Suppl. Table 6). No changes were detected in spinophilin protein levels at P40 (*p* > 0.05; Suppl. Fig. 1c, d). Thus, the observed LPS-induced changes in gene and protein expression at P6 do not remain into adolescence.

#### Neonatal Challenge with LPS and Hypoxia Does Not Induce Grey or White Matter Lesions in Adolescent Male Mice

**Fig. 5:**
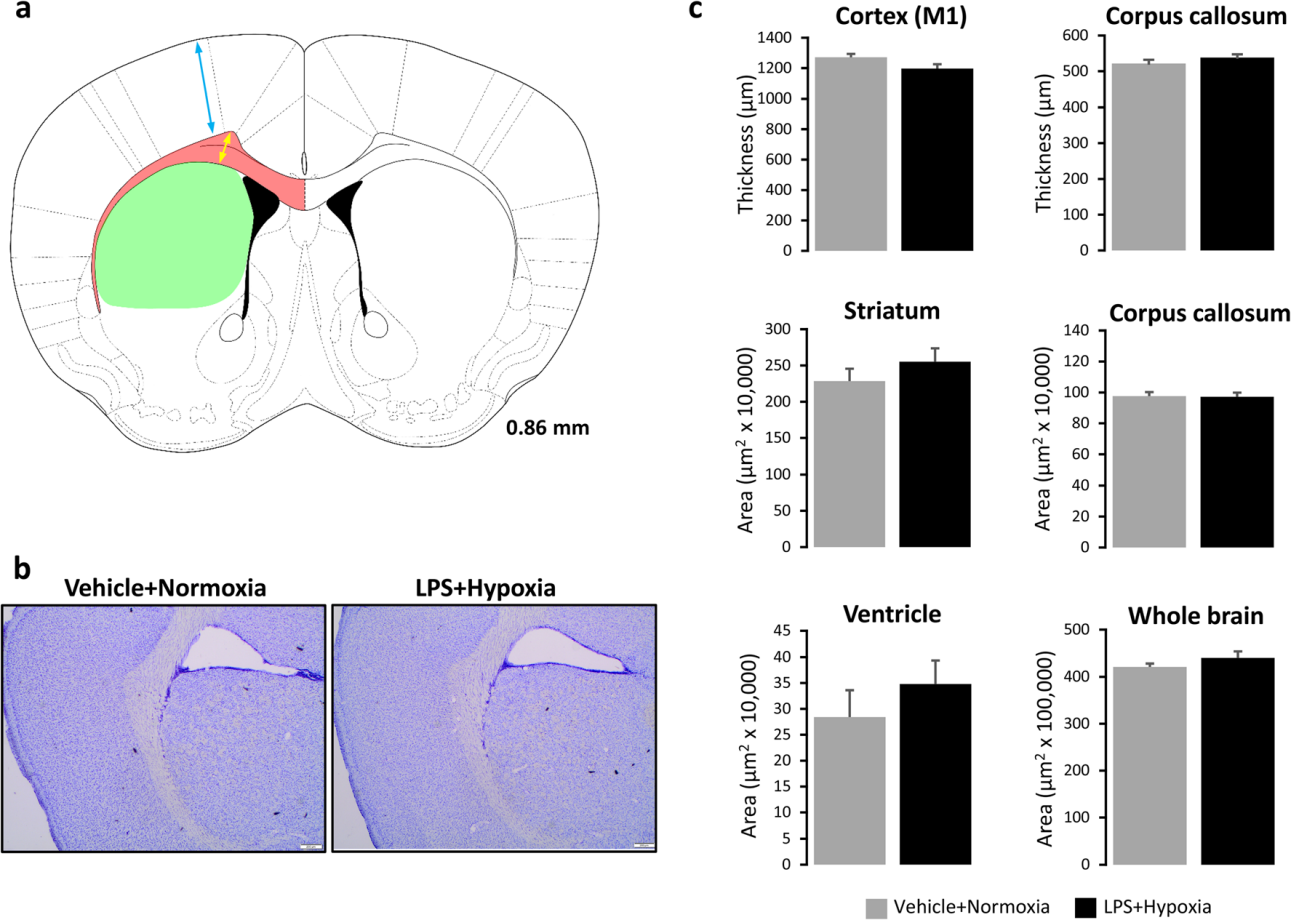
Histological analysis of the brains of 40-day-old male mice. **a** Drawing illustrating the location where histological measurements were obtained. Coronal sections are marked in millimetres from the bregma according to the Mouse Brain atlas of [50]. Arrows and areas in colour correspond to thickness and area measurements to the following brain regions: primary motor cortex (M1) (light blue), corpus callosum (yellow, pink) and striatum (light green). Ventricles are labelled in black. **b** Representative images of cresyl violet staining performed on 20-μm-thick cryosections from P40 mice neonatally exposed to Vehicle + Normoxia or LPS + Hypoxia. No infarcts or lesions were observed. **c** Thickness (μm) and/or area measurements (μm^2^) of M1, corpus callosum and striatum regions, whole brain and ventricles. Bar graphs show means ± SEM (*n* = 6/group). Scale bars: 200 μm. Abbreviation: *LPS* lipopolysaccharide

**Fig. 6:**
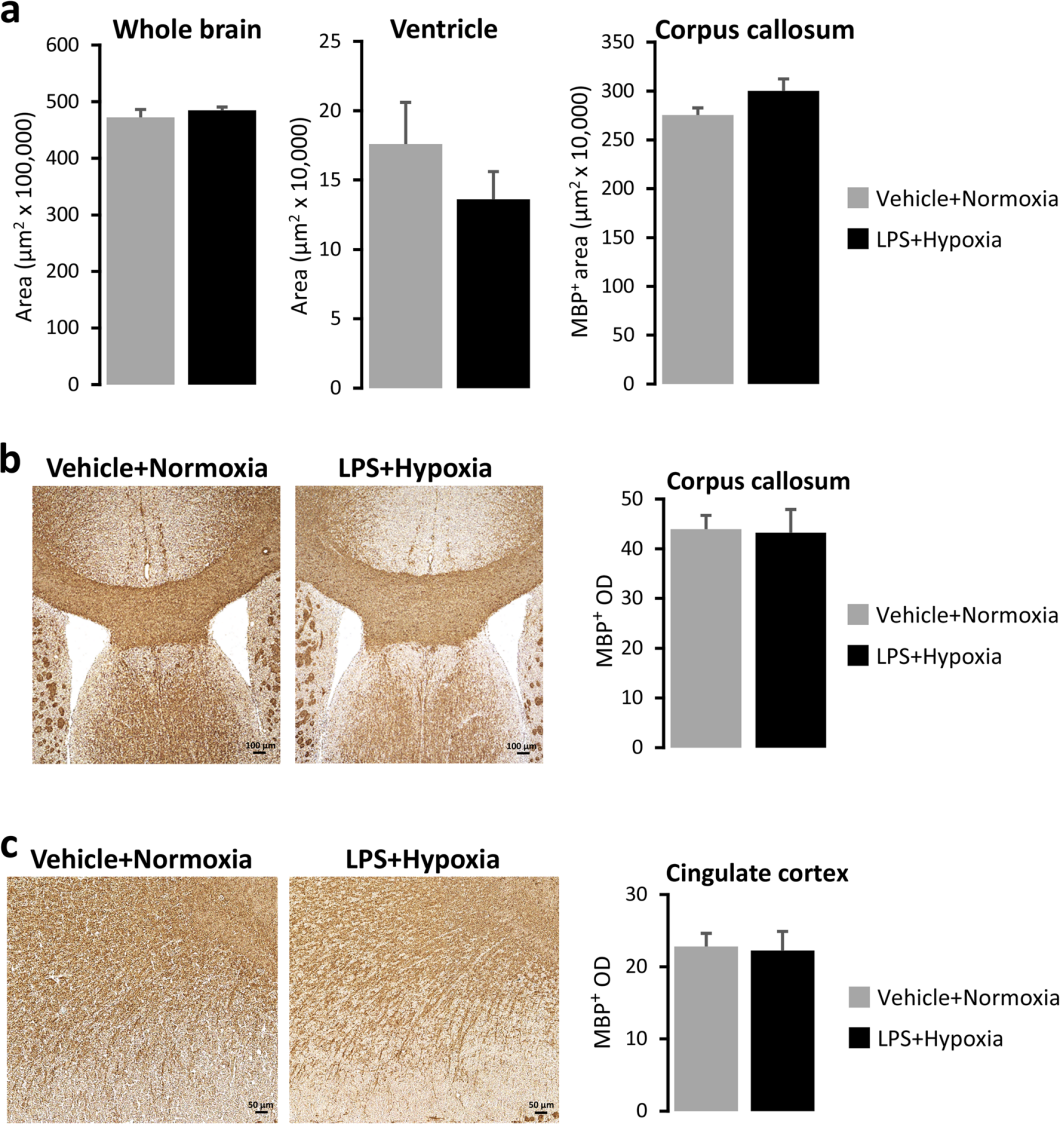
Immunohistological analysis of the brains of 50-day-old male mice. **a** Area measurements (μm^2^) of whole brain, ventricles and corpus callosum. **b**, **c** Representative images of corpus callosum (**b**) and cingulate cortex (**c**). MBP positive staining performed on 10-μm-thick cryosections from P50 mice neonatally exposed to Vehicle + Normoxia or LPS + Hypoxia. Bar graphs show means ± SEM of MBP positive optical density (OD) (*n* = 6/group). Scale bars: 100 μm (**b**), 50 μm (**c**). Abbreviation: *LPS* lipopolysaccharide

Histological examination of the brains of 40-day-old male mice (from cohorts B1 and B2) neonatally exposed to LPS and hypoxia did not reveal any infarcts or lesions compared to their controls (Fig. 5a, b). Moreover, there were no significant differences in the thickness of primary motor cortex (M1) and striatum or the area measurements of striatum, corpus callosum, whole brain and ventricles between groups (Fig. 5c). In addition, no differences in area measurements (Fig. 6a) or MBP optical density in corpus callosum (Fig. 6b) and cingulate cortex (Fig. 6e) were observed in the brains of 50-day-old male mice (from cohort B3) neonatally exposed to LPS and hypoxia compared to their controls. So, the neonatal LPS and hypoxia challenge did not cause histomorphological alterations or white matter lesions detectable in the brains of adolescent mice.

#### Neonatal Challenge with LPS and Hypoxia Does Not Cause Long-Term Changes in Microglia

**Fig. 7:**
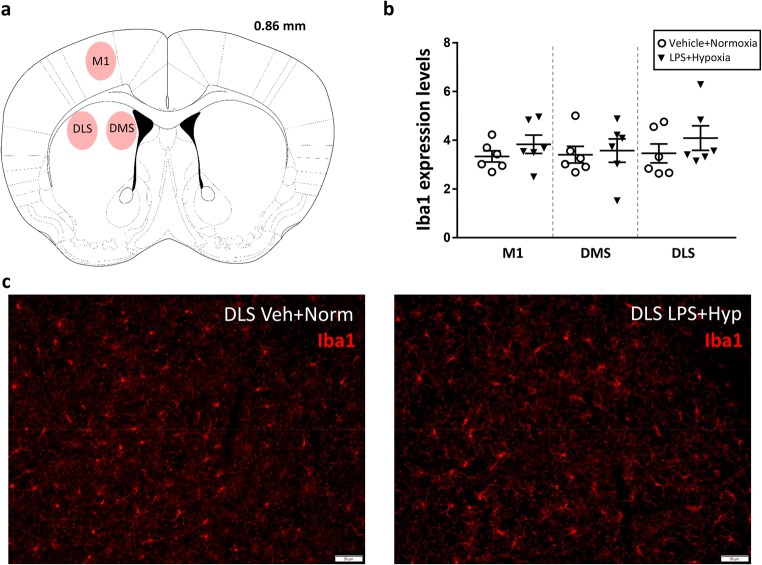
Microglia morphology and density in the primary motor cortex and striatum of adolescent male mice, as judged by ionised calcium binding adaptor molecule 1 (Iba1) immunostaining. **a** Drawing illustrating the location where the IBA1 measurements were obtained. Coronal sections are marked in millimetres from the bregma according to the Mouse Brain atlas of [50]. Labelled areas correspond to the following brain regions: primary motor cortex (M1), dorsomedial striatum (DMS) and dorsolateral striatum (DLS). **b** Scatter plots showing means (± SEM) and individual values of IBA1 expression levels in M1, DMS and DLS areas of control and exposed mice (*n* = 6/group). **c** Representative images of 20-μm-thick IBA1 immunostained brain sections from one control and one exposed mouse. Scale bars: 50 μm. Abbreviations: *Veh* vehicle, *Norm* normoxia, *LPS* lipopolysaccharide, *Hyp* hypoxia

There were no significant changes in the morphology or density of microglia, as assessed by Iba1 immunostaining, in the primary motor cortex (M1) or striatum [dorsomedial (DMS) and dorsolateral (DLS)] of adolescent (40-day-old) male mice neonatally exposed to LPS and hypoxia when compared to their respective controls (Fig. 7). This observation implies that neonatal challenge with LPS and hypoxia did not induce long-lasting microglia activation in the adolescent mice, at least not in the brain regions studied.

## Discussion

Here, we demonstrated that early postnatal exposure of mice to an immunogenic substance (LPS), in combination with hypoxia, leads to subtle motor impairments, which partly reflect gross motor and fine motor impairments in children with CP. The main motor symptom found in both male and female mice was impaired limb placement when walking on a horizontal ladder. Males also exhibited impaired gait, and deficits in fine motor skill learning (females were not tested), while females showed reduced forelimb muscle strength. Neither male nor female mice showed any impairments on the rotarod, indicating that gross motor function and balance were mainly intact. No consistent changes in exploratory/anxiety-related behaviour or memory function were detected in the exposed animals.

The relatively mild CP-like impairments are consistent with the absence of pathological findings in the brains of the adolescent mice. There were neither grey nor white matter lesions, which are common in children with CP [52], and there was no ventricle enlargement. In addition, no chronic brain inflammation was observed, in contrast to other studies that have shown prolonged expression of cytokines, especially of IL1b, after neonatal infection [53]. However, about 10% of children with CP have no detectable brain lesions on magnetic resonance imaging (MRI) scans [38], indicating that the motor symptoms can develop without macroscopic brain injuries. Previous animal studies using exposure to both inflammation and hypoxia have shown long-term morphological changes, including white matter lesions, delayed cortical myelination, microglia activation and astrogliosis, but no consistent pattern regarding effects on anxiety, locomotion, gait and gross motor function. In all the cases, however, the systemic inflammation was induced prenatally and the hypoxia at birth or postnatally [29, 31, 54], or both insults were induced prenatally [55]. Only one study used the combination of systemic inflammation and hypoxia postnatally, but it focused only on neuronal excitability [30].

In agreement with our behavioural observations, Stigger and colleagues reported motor impairments in the walking ladder test after prenatal exposure to LPS, perinatal anoxia and/or sensorimotor restriction from P2 to P28 in rats, which were more pronounced when more than two insults were combined [31]. In addition, no sign of motor impairment was shown using the rotarod and/or the narrow beam after perinatal anoxia [56–58] or LPS [39]. Similarly, exposure to hypoxia-ischaemia (HI) during the neonatal period resulted in long-term sensorimotor dysfunction, hind limb paresis, incoordination and other motor deficits [34, 59, 33, 18, 60, 61]. A previous study by Hu and colleagues also found changes in the gait pattern after prenatal LPS exposure. In contrast to the present results, these authors found larger footprint repeat space between the forelimbs and hindlimbs of rats after exposure [55]. These discrepancies could be due to the time of LPS exposure (prenatal vs postnatal).

The worse performance of the exposed males in the skilled reaching task (SRT) suggests that the developing cortical-striatal circuits involved in motor skill learning are particularly sensitive to perinatal insults [11]. However, this should be confirmed in future studies using larger numbers of animals. So far, the SRT paradigm has only been used for rehabilitation purposes in stroke models (HI in adult animals) [62] and only in one study in young animals [18].

With regard to the exploratory activity and/or anxiety-like behaviour, the observed increased rearing activity of exposed males during the novelty phase of the open-field testing indicates increased exploratory activity [63]. This interpretation is consistent with findings from the EPM test, where the exposed males spent significantly more time in the open arms. Hence, increased anxiety was not detected in the exposed males. In contrast, the exposed females were more anxious in the open field compared to their controls. They also showed traits of anxiety in the LD task. The suggested anxiety phenotype, however, was not confirmed in the EPM task, so it was mild or task-dependent. Importantly, these results were not due to motor deficits in the exposed mice because the overall distance moved was similar for both the exposed and the control groups. Systemic inflammation induced with LPS is well known to induce an anxiety phenotype, but this has not been previously investigated in combination with hypoxia [19, 64]. Nevertheless, there are also studies showing no effects on anxiety after prenatal LPS exposure [35].

Within the cognitive domain, the lack of memory deficits in the present study was surprising since perinatal infection and hypoxia are generally considered as high-risk factors for developing cognitive dysfunction in humans [65, 55]. Favrais and collaborators found deficits in recognition and spatial memory in adolescent mice after neonatal exposure to IL1b [21]. We also expected to find memory deficits in the adolescent mice since there was a robust increase of *Il1b* and a decrease of *Bdnf* in the hippocampus at P6. It has been previously proposed that the early elevation of cytokines affects primarily the hippocampus [66] and that IL1b interferes with the neuroprotective actions of BDNF in this brain region [67]. It is important to acknowledge though that the object recognition task is only partly controlled by the hippocampus, and this could also explain why the early inflammatory response in the hippocampus did not lead to recognition memory deficits in the long term [68, 69]. Another reason could be the inter-trial interval that we chose, which corresponded to short memory effects [70]. A longer interval may have given a different result.

Importantly, we observed sex- and brain region-dependent changes at the gene expression level and sex-dependent changes at the behavioural level. Similarly, Custodio and colleagues, trying to develop an animal model for autism spectrum disorder, showed that neonatal challenge of mice with LPS induced brain region-specific immune and neurotrophic alterations in the prefrontal cortex, hippocampus and hypothalamus [19], and also sex- and age-specific behavioural alterations. The observation of distinct impairments in exposed male and female mice might be due to a different response of microglia to environmental challenges in a sex- and time-dependent manner (see [71]). Moreover, other studies demonstrate sex differences in white matter damage after LPS exposure, with the damage more pronounced in males [72].

The lack of gross morphological abnormalities, together with the mild CP-like phenotype found in our study, raises several questions about the effect of the brain inflammation and hypoxia. *Was the LPS administration effective?* The first experiment (A) showed that LPS was effective and triggered an intense inflammation in the brain that should make it more susceptible to a subsequent insult [28]. Previous studies have also suggested that acute induction of brain inflammatory responses occurs after maternal infection [73, 74] or neonatal LPS administration in rodents [28, 75, 76]. *Was the hypoxia severe enough to effect the brain?* The mortality rate (18%), indicates that the hypoxia was borderline lethal, resulting probably in circulatory collapse before brain damage. We did not use stronger conditions (e.g. reduced oxygen, longer duration), since pilot experiments showed that this yielded even higher mortality rates in the exposed group. The mortality rate in our study was similar to that observed in the study by Mordel and collaborators (10%) who induced systemic inflammation with IL1b administration from P1 to P6 and then hypoxia (8% O_2_) at P7 [30]. *Was the myelination process affected?* The lack of changes in the expression of the transcription factors *Olig1* and *Olig2*, which are involved in the oligodendrocyte differentiation and myelination [77], suggests that the myelination process was not affected and this could explain the lack of white matter damage. *Were neuroprotective mechanisms induced?* We speculate that the early postnatal (P6) activation of *Tnfa* and the anti-inflammatory cytokine *Il10*, which can exert neuroprotective actions [78, 79], could inhibit the effects of the pro-inflammatory cytokines (e.g. *Il1b*) and protect the immature brain [80, 81]. Supporting this concept, it has been previously suggested that neuroprotective mechanisms are triggered by the combination of inflammation and hypoxia and not by inflammation or hypoxia alone [30, 27]. Furthermore, the dramatic increase of the complement system factors (especially *C3*) might have enhanced brain plasticity through synapse pruning and elimination of injured synapses [82] leading to mild only behavioural changes. Importantly, the peptide *C3a* has been shown to ameliorate behavioural deficits after HI injury, and *C3aR* has been suggested to be a novel therapeutic target for the treatment of neonatal HI encephalopathy [83]. Furthermore, it is worth mentioning the concept of preconditioning here, where a sub-lethal injurious insult can result in a time-dependent protection [27]. More time points of testing could unravel at which age the neonatal double hit would have the strongest impact.

Despite considerable efforts, we are still lacking a validated animal model of CP reflecting the impaired gross and fine motor coordination, or coexisting spasticity or dyskinesia. Spasticity has been difficult to reproduce in animal models [84–87]. A good animal model should depict the brain pathology and reflect the pathophysiology and aetiology of CP [38, 88, 89, 15]. We believe that the development of such animal model may still pose a real challenge, given the important differences between the human and rodent brain in terms of white matter quantity [92], cortical subplate development [93, 32], resilience and degree of plasticity [94, 95].

## Conclusions

The present findings indicate that the neuronal circuitries involved in fine motor control and motor learning are particularly sensitive to the neonatal double hit of systemic inflammation and hypoxia. Our hypothesis is that this double hit orchestrates the activation of genes implicated in both neuroinflammation and plasticity (e.g. pro- and anti-inflammatory cytokines, complement system, trophic factors and dopamine-related genes) in a brain region-specific and sex-dependent manner, leading to mild deficits in motor learning and fine motor skills. Therefore, it will be important to use advanced genetically modified mouse models to unravel the precise molecular mechanisms underlying the observed deficits in skilled motor learning. This information will be useful in the development of new interventions for children and adolescents with CP and other motor disabilities.

## Electronic supplementary material

Suppl. Table 1 Presentation of the name, oligonucleotide (forward and reverse) primer sequence and melting temperature (Tm) of the genes used in the qPCR assay (DOCX 35 kb)

Suppl. Table 2 Gene expression in prefrontal cortex of control (Vehicle) and exposed to LPS (LPS) males and females at postnatal day 6. Values are shown as means ± SEM (*n* = 6/condition/sex). Abbreviations: *Il1b* interleukin 1beta, *Il6* interleukin 6, *Il10* interleukin 10, *Il18* interleukin 18, *Tnfa* tumour necrosis factor alpha, *C1qA* alpha chain of complement C1q subcomponent, *C1qB* beta chain of complement C1q subcomponent, *C3* complement component 3, *Olig1* oligodendrocyte transcription factor 1, *Olig2* oligodendrocyte transcription factor 2, *Mbp* myelin basic protein, *Mog* myelin oligodendrocyte glycoprotein, *Map2* microtubule-associated protein 2, *Bdnf* brain-derived neurotrophic factor, *Syp* synaptophysin, *Ppp1r9b* protein phosphatase 1 regulatory subunit 9B, *PFC* prefrontal cortex, *LPS lipopolysaccharide* (DOCX 35 kb)

Suppl. Table 3 Gene expression in striatum of control (Vehicle) and exposed to LPS (LPS) males and females at postnatal day 6. Values are shown as means ± SEM (*n* = 5–6/condition/sex). Abbreviations: *Il1b* interleukin 1beta, *Il6* interleukin 6, *Il10* interleukin 10, *Il18* interleukin 18, *Tnfa* tumour necrosis factor alpha, *C1qA* alpha chain of complement C1q subcomponent, *C1qB* beta chain of complement C1q subcomponent, *C3* complement component 3, *Olig1* oligodendrocyte transcription factor 1, *Olig2* oligodendrocyte transcription factor 2, *Mbp* myelin basic protein, *Mog* myelin oligodendrocyte glycoprotein, *Map2* microtubule-associated protein 2, *Bdnf* brain-derived neurotrophic factor, *Syp* synaptophysin, *Ppp1r9b* protein phosphatase 1 regulatory subunit 9B, *STR* striatum, *LPS lipopolysaccharide (DOCX 35 kb)*

Suppl. Table 4 Gene expression in hippocampus of control (Vehicle) and exposed to LPS (LPS) males and females at postnatal day 6. Values are shown as means ± SEM (*n* = 6/condition/sex). Abbreviations: *Il1b* interleukin 1beta, *Il6* interleukin 6, *Il10* interleukin 10, *Il18* interleukin 18, *Tnfa* tumour necrosis factor alpha, *C1qA* alpha chain of complement C1q subcomponent, *C1qB* beta chain of complement C1q subcomponent, *C3* complement component 3, *Olig1* oligodendrocyte transcription factor 1, *Olig2* oligodendrocyte transcription factor 2, *Mbp* myelin basic protein, *Mog* myelin oligodendrocyte glycoprotein, *Map2* microtubule-associated protein 2, *Bdnf* brain-derived neurotrophic factor, *Syp* synaptophysin, *Ppp1r9b* protein phosphatase 1 regulatory subunit 9B, *HIP* hippocampus, *LPS lipopolysaccharide (DOCX 35 kb)*

Suppl. Table 5 Gene expression in cerebellum of control (Vehicle) and exposed to LPS (LPS) males and females at postnatal day 6. Values are shown as means ± SEM (*n* = 6/condition/sex). Abbreviations: *Il1b* interleukin 1beta, *Il6* interleukin 6, *Il10* interleukin 10, *Il18* interleukin 18, *Tnfa* tumour necrosis factor alpha, *C1qA* alpha chain of complement C1q subcomponent, *C1qB* beta chain of complement C1q subcomponent, *C3* complement component 3, *Olig1* oligodendrocyte transcription factor 1, *Olig2* oligodendrocyte transcription factor 2, *Mbp* myelin basic protein, *Mog* myelin oligodendrocyte glycoprotein, *Map2* microtubule-associated protein 2, *Bdnf* brain-derived neurotrophic factor, *Syp* synaptophysin, *Ppp1r9b* protein phosphatase 1 regulatory subunit 9B, *CER* cerebellum, *LPS lipopolysaccharide (DOCX 35 kb)*

Suppl. Table 6 Gene expression in prefrontal cortex, striatum, hippocampus and cerebellum of control (Vehicle + Normoxia) and exposed (LPS + Hypoxia) males at postnatal day 40. Values are shown as means ± SEM (*n* = 6/condition). Abbreviations: *Il1b* interleukin 1beta, *Il10* interleukin 10, *Tnfa* tumour necrosis factor alpha, *C1qA* alpha chain of complement C1q subcomponent, *C1qB* beta chain of complement C1q subcomponent, *C3* complement component 3, *Mbp* myelin basic protein, *Mog* myelin oligodendrocyte glycoprotein, *Map2* microtubule-associated protein 2, *Bdnf* brain-derived neurotrophic factor, *Syp* synaptophysin, *Ppp1r9b* protein phosphatase 1 regulatory subunit 9B, *PFC* prefrontal cortex, *STR* striatum, *HIP* hippocampus, *CER* cerebellum, *Veh* vehicle, *Norm* normoxia, *LPS* lipopolysaccharide, *Hyp* hypoxia (DOCX 36 kb)

Suppl. Fig. 1 Protein expression levels of spinophilin in the cerebellum. **a** Representative blots of spinophilin and GAPDH (loading control) from 6-day-old animals (*n* = 4–6/group). **b** Graphs depicting the spinophilin protein fold change per group at P6. **c** Representative blots of spinophilin and GAPDH (loading control) from 40-day-old animals (*n* = 6/group). **d** Graphs depicting the spinophilin protein fold change per group at P40. Bars show means ± SEM. **p < 0.01 when compared to the control group. Abbreviations: *P* postnatal day, *GAPDH* Glyceraldehyde-3-Phosphate Dehydrogenase, *Veh* vehicle, *Norm* normoxia, *LPS* lipopolysaccharide, *Hyp* hypoxia (PDF 400 kb)
