# Supplementary material for: Can Neonatal Systemic Inflammation and Hypoxia Yield a Cerebral Palsy-Like Phenotype in Periadolescent Mice?
Source: Mol Neurobiol. 2019 Apr 2;56(10):6883–900. doi: 10.1007/s12035-019-1548-8 (PMC6728419; doi:10.1007/s12035-019-1548-8)
Supplement: Supplementary file 1 — Presentation of the name, oligonucleotide (forward and reverse) primer sequence and melting temperature (Tm) of the genes used in the qPCR assay (DOCX 35 kb) [file 12035_2019_1548_MOESM1_ESM.docx]

**Supplementary Table 1:** Presentation of the name, oligonucleotide (forward and reverse) primer sequence, and melting temperature (Tm) of the genes used in the qPCR assay

| Gene name | Primer | \| **Sequence 5' to 3'** \| \| --- \| | Tm (^o^C) |
| --- | --- | --- | --- | --- |
| *Il1b* | Forward | GAAGATGGAAAAGCGGTTTG | 50 |
|  | Reverse | GTACCAGTTGGGGAACTCTGC |  |
| *Il6* | Forward | ACACATGTTCTCTGGGAAATC | 50 |
|  | Reverse | AGTGCATCATCGTTGTTCATA |  |
| *Il10* | Forward | CTCCCCTGTGAAAATAAGAGC | 50 |
|  | Reverse | GCCTTGTAGACACCTTGGTC |  |
| *Il18* | Forward | TTCGTTGACAAAAGACAGCC | 50 |
|  | Reverse | TATCAGTCTGGTCTGGGGTTC |  |
| *Tnfa* | Forward | CCGATGGGTTGTACCTTGTCT | 50 |
|  | Reverse | GTGGGTGAGGAGCACGTAGT |  |
| *Olig1* | Forward | TACAGGCAGCCACCTATCTCC | 50 |
|  | Reverse | CGAGGTTCAGCGAGCG |  |
| *Olig2* | Forward | CAGCGGCTTCACAGGAGGGACT | 50 |
|  | Reverse | GGCTGCGGGAGGGAGGAT |  |
| *C1qA* | Forward | CAAGGACTGAAGGGCGTGAA | 60 |
|  | Reverse | CAAGCGTCATTGGGTTCTGC |  |
| *C1qB* | Forward | TACTCCAGGCCCCTCTGGAC | 60 |
|  | Reverse | TTGATGGTCCTCAGGGCAGA |  |
| *C3* | Forward | CCAGCTCCCCATTAGCTCTG | 60 |
|  | Reverse | GCACTTGCCTCTTTAGGAAGTC |  |
| *Syp* | Forward | AAGCAACCAGCACCTGGCGA | 60 |
|  | Reverse | TGGCTGCCCGTAATCGGGTT |  |
| *Ppp1r9b* | Forward | CTCAGAAGCGGACTTGGTAG | 60 |
|  | Reverse | GGTCCTCTTCCTCTGAAAGC |  |
| *Bdnf* | Forward | TGGCTGACACTTTTGAGCAC | 50 |
|  | Reverse | GCCAGTCGGCATCGTTTATG |  |
| *Map2* | Forward | CCAGGTGGTGGACGCGTGAA | 60 |
|  | Reverse | CTTAGCGAGCGCCGCAGTGA |  |
| *Mbp* | Forward | CTGGCCACAGCAAGTACCA | 60 |
|  | Reverse | AAAGAAGCGCCCGATGGA |  |
| *Mog* | Forward | ACGATCCTCCTGCAGGTCT | 60 |
|  | Reverse | TCCTCTCAGTCTGTGCTGC |  |
| *Tbp* | Forward | CTCAGTTACAGGTGGCAGGA | 50–60 |
|  | Reverse | CAGCACAGAGCAAGCAACTC |  |
