# Supplementary material for: Can Neonatal Systemic Inflammation and Hypoxia Yield a Cerebral Palsy-Like Phenotype in Periadolescent Mice?
Source: Mol Neurobiol. 2019 Apr 2;56(10):6883–900. doi: 10.1007/s12035-019-1548-8 (PMC6728419; doi:10.1007/s12035-019-1548-8)
Supplement: Supplementary file 4 — Gene expression in hippocampus of control (Vehicle) and exposed to LPS (LPS) males and females at postnatal day 6. Values are shown as means ± SEM (n = 6/condition/sex). Abbreviations: Il1b interleukin 1beta, Il6 interleukin 6, Il10 interleukin 10, Il18 interleukin 18, Tnfa tumour necrosis factor alpha, C1qA alpha chain of complement C1q subcomponent, C1qB beta chain of complement C1q subcomponent, C3 complement component 3, Olig1 oligodendrocyte transcription factor 1, Olig2 oligodendrocyte transcription factor 2, Mbp myelin basic protein, Mog myelin oligodendrocyte glycoprotein, Map2 microtubule-associated protein 2, Bdnf brain-derived neurotrophic factor, Syp synaptophysin, Ppp1r9b protein phosphatase 1 regulatory subunit 9B, HIP hippocampus, LPS lipopolysaccharide (DOCX 35 kb) [file 12035_2019_1548_MOESM4_ESM.docx]

**Supplementary Table 4.** Gene expression in hippocampus of control (Vehicle) and exposed to LPS (LPS) males and females at postnatal day 6

| **HIP** | | **MALES** | | | | **FEMALES** | | | |
| --- | --- | --- | --- | --- | --- | --- | --- | --- | --- |
| **Gene category** | **Gene name** | **Vehicle** | **LPS** | **U** | ***p***  **value** | **Vehicle** | **LPS** | **U** | ***p***  **value** |
| **Cytokines** | ***Il1b*** | 1.02 ± 0.09 | 4.92 ± 0.79 | 0.000 | **0.008** | 1.00 ± 0.04 | 4.83 ± 0.74 | 0.000 | **0.009** |
|  | ***Il6*** | 1.03 ± 0.11 | 1.24 ± 0.23 | 15.000 | 0.818 | 1.03 ± 0.10 | 1.01 ± 0.07 | 13.000 | 0.818 |
|  | ***Il10*** | 1.03 ± 0.11 | 5.28 ± 1.14 | 0.000 | **0.008** | 1.03 ± 0.10 | 2.75 ± 0.35 | 0.000 | **0.009** |
|  | ***Il18*** | 1.01 ± 0.06 | 1.10 ± 0.09 | 13.500 | 0.705 | 1.00 ± 0.04 | 0.74 ± 0.04 | 1.000 | **0.009** |
|  | ***Tnfa*** | 1.03 ± 0.11 | 1.51 ± 0.17 | 5.500 | 0.109 | 1.02 ± 0.09 | 2.51 ± 0.37 | 0.000 | **0.009** |
| **Complement system** | ***C1qA*** | 1.00 ± 0.04 | 2.35 ± 0.19 | 0.000 | **0.008** | 1.01 ± 0.07 | 1.85 ± 0.20 | 1.000 | **0.009** |
|  | ***C1qB*** | 1.01 ± 0.06 | 2.05 ± 0.21 | 0.000 | **0.008** | 1.02 ± 0.08 | 1.84 ± 0.12 | 0.000 | **0.009** |
|  | ***C3*** | 1.07 ± 0.19 | 15.00 ± 3.12 | 0.000 | **0.013** | 1.02 ± 0.10 | 7.66 ± 1.14 | 0.000 | **0.009** |
| **Preoligodendrocyte factors** | ***Olig1*** | 1.00 ± 0.04 | 1.00 ± 0.06 | 16.000 | 0.818 | 1.00 ± 0.04 | 0.88 ± 0.02 | 2.000 | **0.018** |
|  | ***Olig2*** | 1.00 ± 0.04 | 1.06 ± 0.07 | 16.000 | 0.818 | 1.01 ± 0.05 | 1.18 ± 0.12 | 12.000 | 0.485 |
| **Myelin** | ***Mbp*** | 1.02 ± 0.08 | 0.98 ± 0.11 | 16.000 | 0.818 | 1.03 ± 0.10 | 0.81 ± 0.08 | 9.000 | 0.262 |
|  | ***Mog*** | 1.03 ± 0.11 | 1.20 ± 0.13 | 12.500 | 0.630 | 1.04 ± 0.14 | 1.57 ± 0.36 | 15.000 | 0.799 |
| **Grey matter** | ***Map2*** | 1.00 ± 0.03 | 0.97 ± 0.02 | 15.500 | 0.818 | 1.03 ± 0.11 | 0.81 ± 0.07 | 10.000 | 0.320 |
| **Brain plasticity** | ***Bdnf*** | 1.00 ± 0.04 | 0.84 ± 0.07 | 7.500 | 0.213 | 1.01 ± 0.07 | 0.80 ± 0.05 | 4.500 | **0.046** |
|  | ***Syp*** | 1.01 ± 0.04 | 0.92 ± 0.04 | 9.500 | 0.320 | 1.02 ± 0.09 | 0.85 ± 0.04 | 8.500 | 0.211 |
|  | ***Ppp1r9b*** | 1.00 ± 0.04 | 0.92 ± 0.03 | 8.500 | 0.264 | 1.02 ± 0.09 | 0.94 ± 0.13 | 16.000 | 0.818 |

*Notes:* Vehicle and LPS samples were compared with the non-parametric Mann-Whitney U test within the same sex and data are shown as means±SEM. The *p* values were adjusted for multiple comparisons (among the genes) with the false discovery rate (FDR) method. N=6 animals / condition (Vehicle or LPS) / sex.
