# Supplementary material for: Can Neonatal Systemic Inflammation and Hypoxia Yield a Cerebral Palsy-Like Phenotype in Periadolescent Mice?
Source: Mol Neurobiol. 2019 Apr 2;56(10):6883–900. doi: 10.1007/s12035-019-1548-8 (PMC6728419; doi:10.1007/s12035-019-1548-8)
Supplement: Supplementary file 6 — Gene expression in prefrontal cortex, striatum, hippocampus and cerebellum of control (Vehicle + Normoxia) and exposed (LPS + Hypoxia) males at postnatal day 40. Values are shown as means ± SEM (n = 6/condition). Abbreviations: Il1b interleukin 1beta, Il10 interleukin 10, Tnfa tumour necrosis factor alpha, C1qA alpha chain of complement C1q subcomponent, C1qB beta chain of complement C1q subcomponent, C3 complement component 3, Mbp myelin basic protein, Mog myelin oligodendrocyte glycoprotein, Map2 microtubule-associated protein 2, Bdnf brain-derived neurotrophic factor, Syp synaptophysin, Ppp1r9b protein phosphatase 1 regulatory subunit 9B, PFC prefrontal cortex, STR striatum, HIP hippocampus, CER cerebellum, Veh vehicle, Norm normoxia, LPS lipopolysaccharide, Hyp hypoxia (DOCX 36 kb) [file 12035_2019_1548_MOESM6_ESM.docx]

| **MALES** | | **PFC** | | | | **STR** | | | | **HIP** | | | | **CER** | | | |
| --- | --- | --- | --- | --- | --- | --- | --- | --- | --- | --- | --- | --- | --- | --- | --- | --- | --- |
| **Gene category** | **Gene name** | **Veh+**  **Norm** | **LPS+**  **Hyp** | ***U*** | ***p***  **value** | **Veh+**  **Norm** | **LPS+**  **Hyp** | ***U*** | ***p***  **value** | **Veh+**  **Norm** | **LPS+**  **Hyp** | ***U*** | ***p***  **value** | **Veh+**  **Norm** | **LPS+**  **Hyp** | ***U*** | ***p***  **value** |
| **Cytokines** | ***Il1b*** | 1.01 ± 0.07 | 1.04 ± 0.09 | 16.000 | 0.818 | 1.03 ± 0.11 | 1.04 ± 0.15 | 17.500 | 0.656 | 1.01 ± 0.07 | 1.26 ± 0.17 | 12.000 | 0.525 | 1.00 ± 0.04 | 0.99 ± 0.08 | 14.500 | 0.785 |
|  | ***Il10*** | 1.07 ± 0.18 | 1.27 ± 0.21 | 13.500 | 0.644 | 1.03 ± 0.11 | 1.10 ± 0.23 | 16.000 | 0.892 | 1.04 ± 0.13 | 1.30 ± 0.17 | 8.000 | 0.317 | 1.02 ± 0.09 | 1.21 ± 0.09 | 10.000 | 0.411 |
|  | ***Tnfa*** | 1.00 ± 0.04 | 1.22 ± 0.09 | 5.000 | 0.749 | 1.02 ± 0.09 | 1.07 ± 0.11 | 16.500 | 0.892 | 1.01 ± 0.06 | 1.26 ± 0.11 | 6.500 | 0.195 | 1.00 ± 0.04 | 1.13 ± 0.06 | 8.000 | 0.317 |
| **Complement system** | ***C1qA*** | 1.01 ± 0.06 | 0.81 ± 0.05 | 6.000 | 0.246 | 1.00 ± 0.04 | 1.14 ± 0.06 | 7.000 | 0.531 | 1.00 ± 0.04 | 0.88 ± 0.07 | 10.000 | 0.465 | 1.01 ± 0.07 | 1.02 ± 0.04 | 17.000 | 1.000 |
|  | ***C1qB*** | 1.01 ± 0.07 | 0.70 ± 0.04 | 1.000 | **0.048** | 1.01 ± 0.05 | 1.10 ± 0.05 | 9.500 | 0.531 | 1.00 ± 0.04 | 0.72 ± 0.05 | 1.000 | **0.048** | 1.01 ± 0.07 | 1.01 ± 0.06 | 18.000 | 1.000 |
|  | ***C3*** | 1.03 ± 0.13 | 1.66 ± 0.23 | 5.000 | 0.246 | 1.02 ± 0.10 | 2.12 ± 0.47 | 3.000 | 0.360 | 1.07 ± 0.16 | 1.65 ± 0.38 | 11.000 | 0.465 | 1.11 ± 0.22 | 2.15 ± 0.25 | 4.000 | 0.156 |
| **Myelin** | ***Mbp*** | 1.12 ± 0.08 | 0.81 ± 0.07 | 3.500 | 0.180 | 1.00 ± 0.04 | 1.07 ± 0.11 | 14.500 | 0.785 | 1.01 ± 0.07 | 0.89 ± 0.11 | 13.000 | 0.582 | 1.00 ± 0.04 | 1.13 ± 0.05 | 8.000 | 0.317 |
|  | ***Mog*** | 1.03 ± 0.10 | 0.88 ± 0.04 | 10.500 | 0.480 | 1.01 ± 0.05 | 1.18 ± 0.10 | 11.000 | 0.531 | 1.02 ± 0.09 | 1.01 ± 0.13 | 17.000 | 0.937 | 1.01 ± 0.06 | 1.12 ± 0.05 | 10.500 | 0.411 |
| **Grey matter** | ***Map2*** | 1.03 ± 0.09 | 0.94 ± 0.04 | 11.000 | 0.531 | 1.08 ± 0.19 | 1.29 ± 0.04 | 11.000 | 0.531 | 1.01 ± 0.06 | 0.82 ± 0.05 | 5.000 | 0.164 | 1.01 ± 0.05 | 1.08 ± 0.04 | 12.000 | 0.591 |
| **Brain plasticity** | ***Bdnf*** | 1.02 ± 0.07 | 1.15 ± 0.05 | 8.500 | 0.317 | 1.29 ± 0.39 | 1.44 ± 0.37 | 17.000 | 1.000 | 1.00 ± 0.03 | 0.83 ± 0.03 | 2.000 | 0.054 | 1.00 ± 0.04 | 1.35 ±0.05 | 0.000 | **0.048** |
|  | ***Syp*** | 0.97 ± 0.01 | 0.98 ± 0.08 | 11.000 | 0.644 | 1.04 ± 0.14 | 1.19 ± 0.13 | 11.000 | 0.531 | 1.00 ± 0.04 | 0.93 ± 0.03 | 11.000 | 0.465 | 1.00 ± 0.04 | 0.91 ± 0.03 | 8.000 | 0.317 |
|  | ***Ppp1r9b*** | 1.01 ± 0.05 | 0.98 ± 0.07 | 10.500 | 0.644 | 1.04 ± 0.15 | 1.39 ± 0.04 | 6.000 | 0.531 | 1.01 ± 0.06 | 1.05 ± 0.07 | 12.000 | 0.722 | 1.01 ± 0.06 | 1.01 ± 0.04 | 13.500 | 0.950 |

**Supplementary Table 6.** Gene expression in prefrontal cortex, striatum, hippocampus and cerebellum of control (Vehicle+Normoxia) and exposed (LPS+Hypoxia) males at postnatal day 40

*Notes:* Control and exposed samples were compared with the non-parametric Mann-Whitney U test within the same sex (males only) and data are shown as means±SEM. The *p* values were adjusted for multiple comparisons (among the genes) with the false discovery rate (FDR) method. N=6 animals / condition (Vehicle or LPS) / sex. Abbreviations: *Veh* vehicle, *Norm* normoxia, *LPS* lipopolysaccharide, *Hyp* hypoxia
