# Supplementary figures and images for: Can Neonatal Systemic Inflammation and Hypoxia Yield a Cerebral Palsy-Like Phenotype in Periadolescent Mice?
Source: Mol Neurobiol. 2019 Apr 2;56(10):6883–900. doi: 10.1007/s12035-019-1548-8 (PMC6728419; doi:10.1007/s12035-019-1548-8)

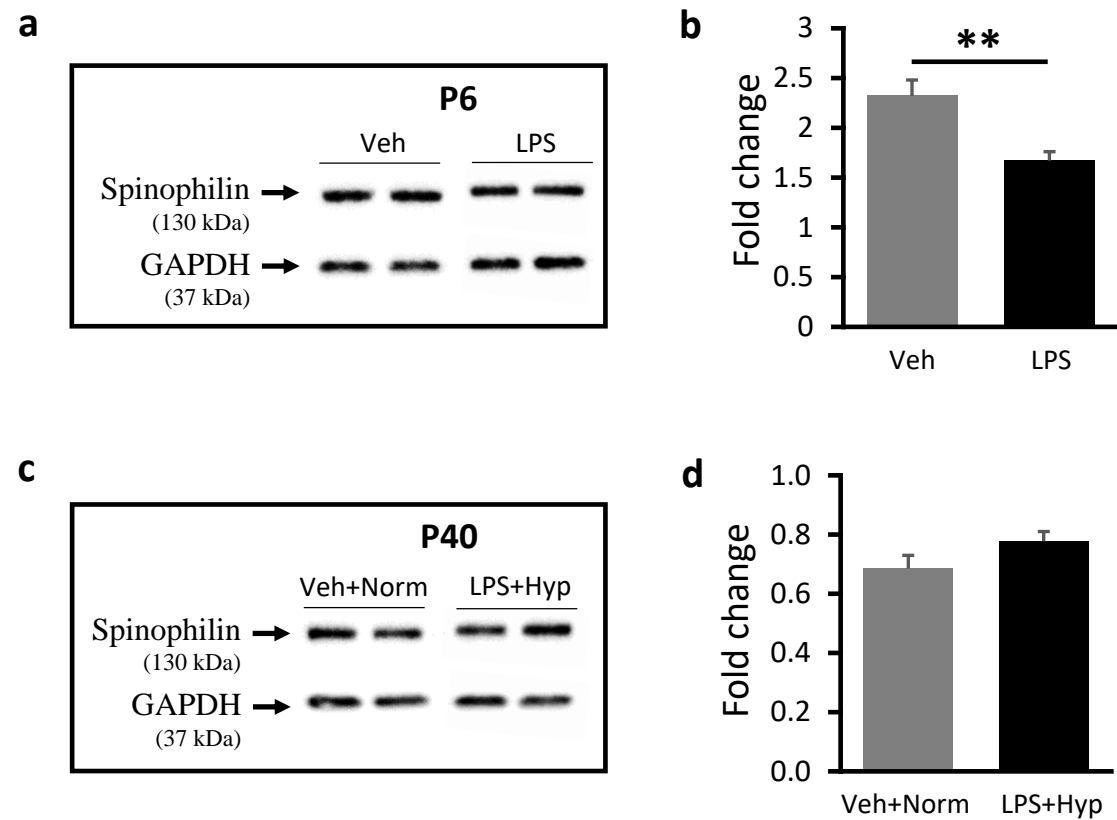

Supplementary Figure 1

Supplement: Supplementary file 7 — Protein expression levels of spinophilin in the cerebellum. a Representative blots of spinophilin and GAPDH (loading control) from 6-day-old animals (n = 4–6/group). b Graphs depicting the spinophilin protein fold change per group at P6. c Representative blots of spinophilin and GAPDH (loading control) from 40-day-old animals (n = 6/group). d Graphs depicting the spinophilin protein fold change per group at P40. Bars show means ± SEM. **p < 0.01 when compared to the control group. Abbreviations: P postnatal day, GAPDH Glyceraldehyde-3-Phosphate Dehydrogenase, Veh vehicle, Norm normoxia, LPS lipopolysaccharide, Hyp hypoxia (PDF 400 kb) [file 12035_2019_1548_MOESM7_ESM.pdf]
